# In Vitro and In Vivo Cytotoxicity of Boron Nitride Nanotubes: A Systematic Review

**DOI:** 10.3390/nano12122069

**Published:** 2022-06-15

**Authors:** Akesh Babu Kakarla, Ing Kong

**Affiliations:** School of Computing, Engineering and Mathematical Sciences, La Trobe University, Bendigo, VIC 3552, Australia; a.kakarla@latrobe.edu.au

**Keywords:** boron nitride nanotubes, toxicity, cytotoxicity, biocompatibility, biomedical, tissue engineering

## Abstract

Boron nitride nanotubes (BNNTs) are an exciting class of nanomaterials due to their unique chemical and physical characteristics. In recent decades, BNNTs have gained huge attention in research and development for various applications, including as nano-fillers for composites, semiconductor devices, hydrogen storage, and as an emerging material in biomedical and tissue engineering applications. However, the toxicity of BNNTs is not clear, and the biocompatibility is not proven yet. In this review, the role of BNNTs in biocompatibility studies is assessed in terms of their characteristics: cell viability, proliferation, therapeutic outcomes, and genotoxicity, which are vital elements for their prospective use in biomedical applications. A systematic review was conducted utilising the databases Scopus and Web of Science (WOS) (2008–2022). Additional findings were discovered manually by snowballing the reference lists of appropriate reviews. Only English-language articles were included. Finally, the significant analysis and discussion of the chosen articles are presented.

## 1. Introduction

**Figure 1:**
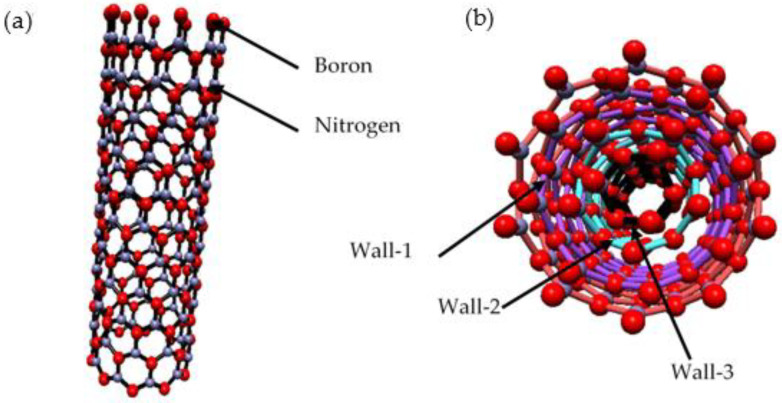
Illustration of (**a**) BNNTs (red—boron, blue—nitrogen); (**b**) multiwalled BNNTs.

The past decade has witnessed the rapid development of nanoscale science and technology that led to the discovery of various interesting elements of boron nitride nanotubes (BNNTs). Since BNNTs were reported theoretically in 1994 [1,2] and produced experimentally in the following year (using an arc discharge method) [3], there has been a lot of interest in the research and development of BNNTs as a counterpart nanomaterial for carbon nanotubes (CNTs). BNNTs are similar in structure to CNTs, being cylindrical rolls in which carbon atoms are altered with boron and nitrogen atoms arranged in a hexagonal lattice (Figure 1) [4]. Consequently, various methods focusing on the synthesis of BNNTs have been developed, such as chemical vapour deposition (CVD) [5], ball milling [6], substitution reactions [7], co-precipitation, and annealing processes [8]. These methods produced various geometric nanotubes, purity, and structures of BNNTs to meet the required physiochemical properties. However, standardised methods to produce high yield and high purity BNNTs is still in early stages [9]. Therefore, the synthesis of BNNTs still appears in literature using different catalyst materials to produce application compatible BNNTs [10,11]. Furthermore, BNNTs are structural analogues of CNTs but possess unique chemical and physical properties. BNNTs are electrical insulation with a wide bandgap (~6.0 eV) [4,12] and the conducting of the tubes is independent of chirality, unlike CNTs [4,12]. In addition, BNNTs possess Young’s modulus up to 1.2 TPa [13] and are stable in air, up to 1100 °C [14]. In comparison, CNTs are chemically stable up to 500 °C in air [12,14]. The thermal conductivity of BNNTs is slightly higher than CNTs (~300 W·mK^−1^) [12,15] at about ~350 W·mK^−1^ [12] for a diameter of tube ranging from 30–40 nm [4]. In addition, the high-purity BNNTs possess an optical band gap of ~6.0 eV with the absorption peak at ~205 nm [12,16]. In addition, it was reported that bending of individual BNNTs can alter the insulating behaviour and act as semiconductors [12,16,17]. Owing to their intriguing physical and chemical properties, BNNTs gained significant interest in various applications such as magnetorheological devices [12], nanoelectronics [18], microelectromechanical systems, space travel [19], optical [20], drug delivery [21], polymer nanocomposite [18], and tissue engineering [18,22].

Despite BNNTs being a promising material for various applications, the hydrophobic nature of pristine BNNTs hindered its exploitation in BNNTs applications. In terms of biomedical applications, the solubility, homogeneity, and stability in aqueous media are vital factors [23]. Therefore, to overcome the challenges, researchers have explored the functionalisation of BNNTs (f-BNNTs) with various organic and inorganic materials to obtain water-soluble BNNTs and to enhance the cellular uptake of BNNTs in biomedical applications [23]. Hence, it was evident that a breakthrough in the synthesis and functionalisation of BNNTs would open doors to employ BNNTs in various biomedical applications. However, the research and development of BNNTs is still in its infancy as regards utilising BNNTs as a mainstream nanomaterial in biomaterial applications. This is because no standardised protocol exists to assess the toxicity and biocompatibility of BNNTs. Considering this, the interaction of BNNTs with various types of cells, tissues, and organs needs to be assessed to identify the toxicity of the material. The adverse effects of foreign materials in clinical applications can impact the normal functions of tissues or organs, which can lead to health issues and ultimately can be lethal to the tissues. 

In this systematic review article, information regarding the toxicity or cytotoxicity as well as the biocompatibility of BNNTs with various cell lines and animals is reported. Indeed, the objective of the review is to clarify the biocompatibility and to promote the design of future BNNTs in biological domains as having potential for tissue-engineering applications.

## 2. Methodology

### 2.1. Eligibility Criteria 

In vitro and in vivo studies that used BNNTs to address toxicity and biocompatibility until February 2022 from Scopus and Web of Science (WOS) databases were included in this review. Included published studies were limited to English language and journal articles only. The search excluded abstracts, reviews, letters, and theses. 

### 2.2. Types of Interventions 

Studies that were conducted using BNNTs as the source to investigate cell viability, cytokines, inflammation, genotoxicity, and toxicity effects on animals were included. Studies that used derivates of boron nitride, boron nitride nanospheres, boron nitride nanoribbons, or boron nitride nanoplates were excluded. Studies discussing density functional theory (DFT) or theoretical BNNT cellular dynamics were also excluded.

### 2.3. Information Source

A systematic search using Scopus and WOS was performed to identify studies (looking at BNNTs in vivo and in vitro) reporting the outcomes of toxicity, cytokines, and biocompatibility of materials. The keywords that identified the studies are listed below (Table 1). All the selected articles’ bibliographies were screened manually. 

### 2.4. Data Collection 

**Figure 2:**
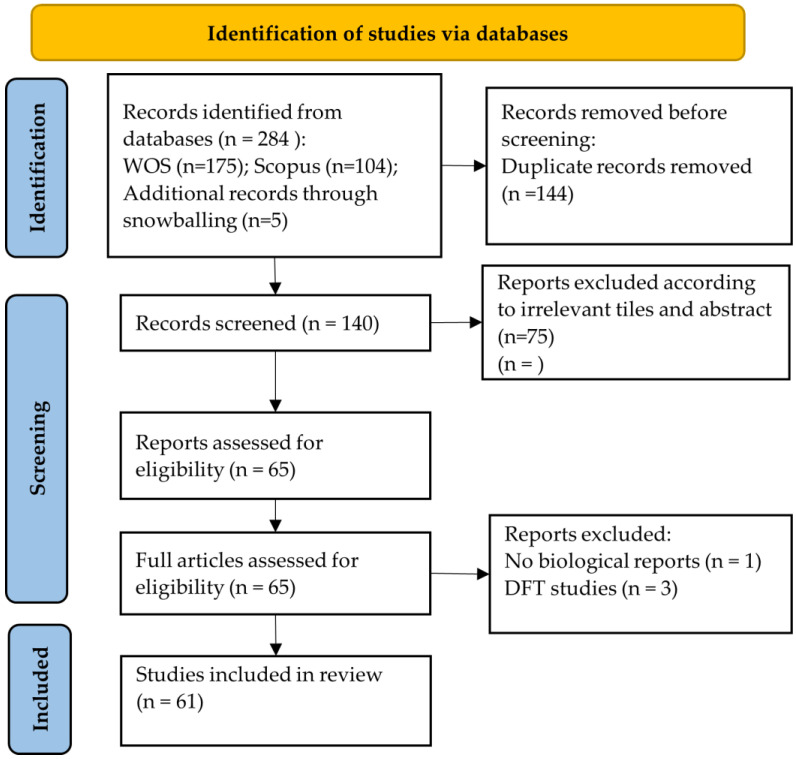
PRISMA search strategy.

The obtained articles from search sources were extracted into an Excel spreadsheet, and this was performed using the PRISMA search strategy (Figure 2). Two independent reviewers performed the screening of the articles. Titles and abstracts were screened initially by one reviewer (A.B.K.) using the selection criteria described in Figure 1. Selected studies from the first screening were then screened independently by two reviewers (A.B.K. and I.K.). The full text of the studies was verified independently by the same reviewers (A.B.K. and I.K.) using the same selection criteria. The reviewers discussed differences in opinion until a consensus was reached. Snowballing search from other reviews and selected papers was also conducted to identify additional articles.

## 3. Results

### 3.1. Study Selection 

The process of article selection and data extraction is shown in Figure 1. In the primary search, a total of 284 articles were found from the Scopus and WOS databases. A total of 140 articles were chosen after duplicates were removed. Then, records were screened and irrelevant studies such as simulations and non-toxicity studies (according to the abstracts and titles) were removed, and 65 studies remained. In the next step, the full texts of 65 selected articles were reviewed, and 4 articles were removed. Ultimately, a total of 61 articles were included in this systematic review (Table 2). 

### 3.2. The Synthesis of BNNTs

The synthesis process of BNNTs essentially depends on the conversion of B and N atoms into BN radicals [84]. Various methods to develop BNNTs depend considerably on diverse strategies, including precursors, conditions, and equipment, to obtain the high-yield BNNTs. In 1995, BNNTs were synthesised along with the analysis of their structural characteristics. The synthesis was carried out using various methods, such as laser ablation [15], chemical vapour deposition (CVD) [16], ball milling [17], substitution reaction [18], co-precipitation, and annealing [19]. All these syntheses produced BNNTs with a variety of purity levels, structures, and diameters to meet the requirements for particular physical and chemical properties. The real applications of BNNTs are still far from the market, but some laboratory-level exploration has been successfully achieved. For example, BNNTs unitisation in various applications such as BNNTs reinforced polymeric composites [20], BNNTs reinforced ceramic composites [21] and hydrogen storage [22] have been carried out effectively.

#### 3.2.1. Arc Discharge Method 

Chopra et al. [3] were the first to report the experimental synthesis of BNNTs by using the arc discharge method. The procedure involved a BN rod as the precursor, which was inserted into a hollow tungsten anode electrode and arc plasma was generated between the precursor and anode to produce BNNTs. The BNNTs produced were multi-layer nanotubes with lengths of 200 nm and a diameter ranging from 1 to 3 nm [3]. The yield ratio of the obtained BNNTs was 1:1 for B:N [3]. Cumings et al. [85] reported the plasma-arc method, which can produce large-scale amounts of pure BNNTs. During the process, a grey, web-like material grew near the top of the chamber, while a thin layer of grey soot covered the side walls of the chamber [85]. Both the web-like material and the grey soot contained high amounts of BNNTs. Nevertheless, the web-like material had a slightly higher amount of BNNTs compared to the grey soot [85]. Saito et al. [86] reported BNNTs synthesised using arc discharge with the reaction between the zirconium diboride electrode in a nitrogen (N_2_) atmosphere. Single-wall BNNTs, with a diameter ranging from 2 to 5 nm, were obtained with different chiral angles containing the zigzag and the armchair [86]. Recently, Yeh et al. [87] demonstrated a stable synthesis of BNNTs, with a precursor of B anode in an atmosphere of N_2_, using the arc discharge method. The report indicated that BNNTs produced from this method were single and multi-walled nanotubes. However, the major drawback of the arc discharge method is that it is challenging to manufacture BNNTs at commercial quantity, as the reaction zone at the arc core is limited to a modest capacity [84].

#### 3.2.2. Ball Milling Method 

Ball milling is another method utilised to synthesise BNNTs [88]. Chen et al. [89] demonstrated the synthesis of BNNTs with a boron powder ball milled under ammonia (NH_3_) gas for 150 h and subsequently annealed at 1000 to 1200 °C in the N_2_ environment. The report stated that the long hours of milling through the nitration process resulted in high-yield BNNTs. Gerald et al. [90] described the BNNTs produced from precursors (tungsten carbide) in a ball mill. The characterisations of the study stated that thick-walled BNNTs were produced in milling conditions under an ammonia atmosphere. Similarly, other researchers produced BNNTs using various catalysts, mainly iron(III) chloride (FeCl_3_) [91], iron (Fe) [6], ferric nitrate (Fe(NO_3_)_3_) [92], iron(II, III) oxide (Fe_3_O_4_) [93], B [94], and silicon carbide (SiC_f_/SiC) [95]. The synthesised BNNTs were mostly in bamboo-like nanotube shapes with a diameter ranging from 50 to 200 nm with a length of 1.0 mm. 

#### 3.2.3. Laser Ablation

The laser ablation method is used to synthesise BNNTs at higher temperatures. Goldberg et al. [96] demonstrated the synthesis of BNNTs through laser heating of precursor cubic-BN and hexagonal-BN in a diamond anvil in the N_2_ atmosphere. The obtained BNNTs were 3–15 nm in diameter. In another study, Yu et al. [97] reported the production of BNNTs using the evaporation of BN, along with cobalt and nickel at 1200 °C in the presence of a laser beam. The findings stated that the BNNTs had a diameter ranging from 1.5 to 8 nm. Additionally, it was further stated that the tips of nanotubes were either flat cap or semi-circular [97]. Smith et al. [98] demonstrated the synthesis of the long single- and multi-walled BNNTs through the pressurised vapour condition. BNNTs of 3 cm in length were obtained. More recently, Kim et al. [99] reported dual growth modes of BNNTs using a high-temperature pressure laser ablation. It was observed that BNNTs with both closed- and open-end nanotubes were obtained [99].

#### 3.2.4. Thermal Plasma

Thermal plasma is another technique used to produce BNNTs. This method is similar to the laser ablation technique but capable of producing a large volume of BNNTs. Kim et al. [100] demonstrated producing BNNTs of small diameter (~5 nm) using hexagonal-BN by the thermal plasma technique. BNNTs with high crystallinity, purity, and yield were produced without needing a higher amount of catalyst [100]. Similarly, Fathalizadeh et al. [101] described a low-wall number BNNTs synthesis method using the thermal plasma method. The result showed that catalyst-free BNNTs with a production rate of 35 g·h^−1^ was obtained [101]. Compared with the laser ablation method, the production rate is 300 times higher than the thermal plasma process [101]. Recently, Kim et al. [102] reported that BNNTs were synthesised using direct current thermal plasma. The report stated that the high-yield BNNTs were produced at a production rate of 12.6 g·h^−1^. BNNTs with a diameter of 7 nm were obtained with low input power and gas. Hence, thermal plasma provides an efficient way to mass-produce BNNTs with high quality [102,103,104]. 

#### 3.2.5. Chemical Vapour Deposition (CVD) and Thermal Annealing

The CVD method has been playing a promising role in synthesising BNNTs. In this process, the yield and structure of the BNNTs are mainly based on the precursor, catalyst, temperature, gas, and equipment. Experimentally, BNNTs grown in the CVD method are commonly through horizontal or vertical furnaces at high temperatures. Furthermore, the furnace with dual temperatures was also considered in the synthesis of BNNTs using CVD. Lee et al. [105] applied the CVD method with a precursor loaded in a quartz tube and placed the tube in a horizontal furnace. The design was used to grow the BNNTs through flowing NH_3_ and react with the precursor to obtain high BNNTs. Recently, Koken et al. [10] reported the synthesis of BNNTs at a temperature of 1050 °C with the precursor (colemanite) and catalyst (Fe_2_O_3_) through CVD. The produced BNNTs were multi-walled with a diameter of 62 to 82 nm. The CVD technique is studied with various precursors, catalysts, and temperatures to produce BNNTs [106]. 

In addition to the methods mentioned above, there are other synthesis techniques to produce BNNTs; for example, the co-precipitation and annealing method, where B powder, Fe_2_O_3_, and urea were mixed to obtain a precursor and annealed at 1200 °C for 5 h [8]. The grown BNNTs had a diameter ranging from 10 to 80 nm with the shape of bamboo and quasi-cylindrical [8]. Another method was heating the B powder, Fe_2_O_2_, and ammonium chloride in the autoclave at 600 °C for 12 h [106,107]. The diameter of obtained BNNTs was 100 nm. To better understand these techniques, they are summarised in Table 3. 

Overall, the thermal plasma method is currently applicable in the commercial production of BNNTs. The cost of 100 mg of BNNTs is within USD 100 [18]. However, ongoing research and development will continue to obtain more efficient methods to produce higher purity and yield BNNTs to stimulate the availability of the material.

### 3.3. BNNTs Functionalisation, Modification, and Types of Composites 

**Figure 3:**
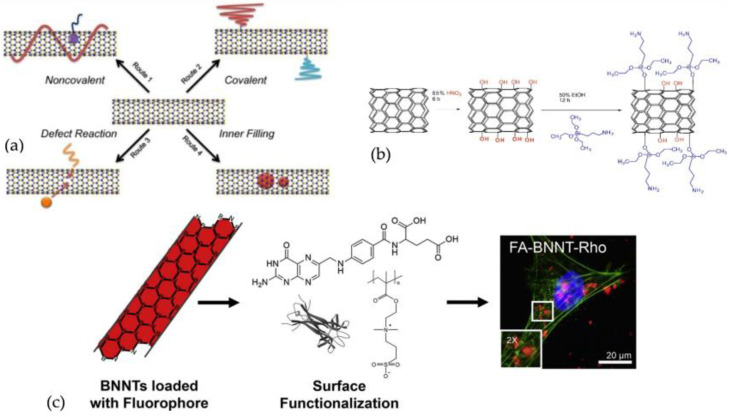
(**a**) Various BNNTs functionalisation methods. Reproduced with permission from Ref. [149]. Copyrights 2014 Sage. (**b**) BNNTs functionalising with -OH groups. Reproduced with permission from Ref. [70]. Copyrights 2012 Elsevier. (**c**) Illustration of BNNTs surface functionalisation and used in the in vitro analysis. Reproduced with permission from Ref. [152]. Copyrights 2020 Elsevier.

Although BNNTs possess excellent characteristics and are recognised as structural analogues of CNTs, the hydrophobic nature of BNNTs limited their utilisation in biomedical applications. One of the major drawbacks of using pristine BNNTs in cell culture is the tendency to form agglomeration [81], which can affect both cell proliferation and biocompatibility evaluation [65,81]. Thus, the initial step to employ BNNTs in biological systems is to obtain the homogenous dispersion of BNNTs in aqueous media or in other physiological media [146,147]. To overcome this limitation, the functionalisation of the surface of BNNTs with various approaches such as covalent [148], noncovalent [149], defect reaction, and inner-space filling [150,151] were employed to give nanotubes a good dispersibility in water and biological mediums (Figure 3a). Additionally, BNNTs were functionalised with lipids [23], antibodies [152], peptides [153], specific molecules, or quantum dots [146] to uncover the full possibilities of using BNNTs in biomedical applications. The illustration of BNNTs functionalisation is shown in Figure 3. 

Experiments were conducted through various consecutive steps of thermal stirring and sonication with various agents such as glycol-chitosan (GC) [76]; poly-L-lysine (PLL) [78]; methoxy–poly(ethylene glycol)–1,2–distearoyl–sn–glycero–3–phosphoethanolamine-N conjugate (mPEG-DSPE) [23]; polyethyleneimine (PEI) [82]; 3-aminopropyltrimethoxysilane (APTES) [70]; doxorubicin (DOX) and deoxyribonucleic acid (DNA), to obtain stable dispersion of BNNTs in cell-culture mediums.

### 3.4. Cell Sources 

The most dominant cell types used in the included articles were human embryonic kidney cells (HEK293, HEK293T) [24,25,74,81,154], human neuroblastoma cells (SH-SY5Y) [30,64,68,73,76,79,82,155], mouse embryonic fibroblasts (NIH/3T3) [70], human dermal fibroblasts (HDF) [31,36,48,51,55], lung fibroblasts (MRC-5) [65,156], dental pulp fibroblasts [34,41,62], lung epithelium cells (A549) [37,55,74], macrophage cells (RAW 264.7) [49,74], human liver epithelium (HeP G2) [27,28,37], human peripheral blood (NB4) [37], glioblastoma cells (malignant U87 [30,37,156], T98 [80,156]), mammary gland adenocarcinoma cells (MCF-7) [156], N9 murine microglia cells [50], osteoblasts [54,66,75,77], osteoblast precursor cell line derived from mouse musculus calvaria (M3CT3) [47], THP-1 [45], NLRP3-deficient human monocytic cells [45], human osteosarcoma cell line (SAOS-2) [31,39], murine macrophages [77], Henrietta Lacks (HeLa) [35,42,43,58], CD34^+^ cells [35], V79 cells [35], bronchoalveolar lavage cells (BAL) [29], B16 melanoma [59], human LNcap prostate cancer cells [61], human endothelial cells (HUVECs) [64,67], human hepatocytes (L02) [27], C2C12 mouse myoblasts [36,56,78,155], human tendon cells [38], 4T1 tumour cells [32], bone-marrow mesenchymal stem cells (MSCs) obtained from bilateral femora from Fischer 344/N syngeneic rats [52], MSCs [60], human prostate adenocarcinoma cells (DU145) [44], MDA-MB-231-luc2 cells [46], and human keratinocytes (HaCaT) [33] to assess the toxicity and biocompatibility of the BNNTs. 

### 3.5. Methods Evaluating In Vitro Biocompatibility of BNNTs

#### 3.5.1. Cell Viability Assays

**Figure 4:**
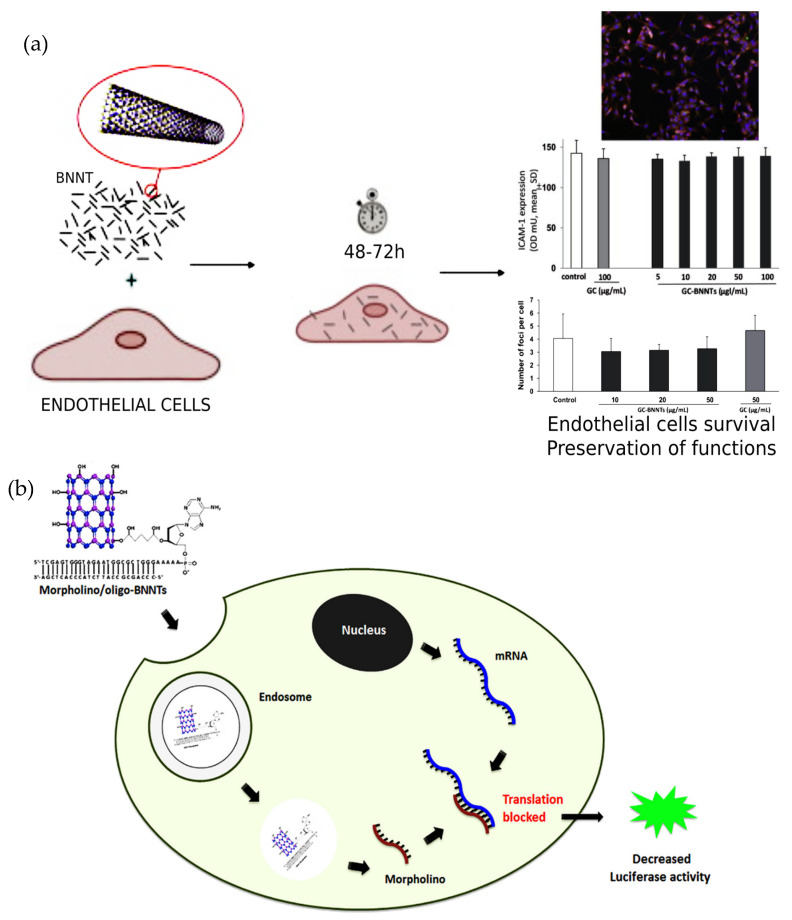
(**a**) Illustration of cell viability assay. Reproduced with permission from Ref. [67]. Copyright 2013 Elsevier; (**b**) Morpholino-oligo-BNNTs cultured with MDA-MB-231-luc2 cells to evaluate gene slicing efficiency. Reproduced with permission from Ref. [46]. Copyright 2017, Elsevier.

In the selected articles various methods have been used to analyse the effects of the BNNTs-incubated cells on cell viability and cytotoxicity assays (Figure 4a). The most commonly used evaluations were 3-(4,5-dimethylthiazol-2-yl)-2,5-diphenyltetrazolium bromide (MTT) [31,35,76,78,82,156], 2-(4-iodophenyl)-3-(4-nitrophenyl)-5-(2,4-disulfophenyl)-2H-tetrazolium (WST-1) [68], alamarBlue™ [30], Trypan Blue assay [78,82], tetrazolium-8-[2-(2-methoxy-4-nitrophenyl)-3-(4-nitrophenyl)-5-(2,4-disulfophenyl)-2H-tetrazolium] monosodium salt (CCK-8) [27], lactic dehydrogenase (LDH), and amido black assay [67]. These assays are calorimetric methods to evaluate cell viability, proliferation, and cytotoxicity. For instance, MTT assays are dissolved in a solubilisation solution that results in a coloured solution with absorbance at 500–600 nm, using a spectrophotometer. Subsequently, WST-1 assay is a water-soluble assay with greater stability and sensitivity, providing rapid measurements. Another method is LDH assay, which is used to evaluate the level of plasma-membrane damage in cell population. Another very simple method is Trypan Blue assay. Trypan Blue highlights the dead cells that are easily observed under bright-field microscopy [24,25,82].

Cytotoxicity of BNNTs can be detected by staining the live and dead cells. The staining reagents penetrate the living cells and transform the membrane into fluorescent dye colours such as blue, green, and red, which can be detected with flow cytometry and fluorescence microscopy [146,157]. To identify the BNNTs cytotoxicity, the studies used calcein AM [31,68], ethidium homodimer III (Ethd-III) [68], annexin V-Fluorescein (FITC)/PI [27,42,49,64,66,76] assay, actin staining [29] and 4′,6-diamidino-2-phenylindole (DAPI) staining and trizima solution [33].

#### 3.5.2. Total Reactive Oxygen Species

Generally, BNNTs (foreign matter) incubated with cells tend to increase reactive oxygen species (ROS) [40,45,64,65,66,76]. The inclined ROS levels cause cellular stress that can be observed using oxidation-sensitive fluorescent dyes like 5-(and-6)-carboxy-2′,7′-dicholrodihydrofluorescein diacetate (carboxy-H2DCFDA) [157]. The evaluation is carried out by flow cytometry in the FITC channel and fluorescence microscopy. The oxidative stress may increase due to sudden changes in environmental conditions or during infection, which causes damage to cellular proteins, lipids, and DNA that has been associated with inflammation, cancer, and other physiological conditions. 

#### 3.5.3. Genotoxicity Evaluation 

Genotoxicity testing is performed to detect and identify hazards, and to determine the mutation of germ cells and cancer development (Figure 4b) [46]. The genotoxicity of BNNTs can be assessed using the comet assay [35,40,57]. The comet assay can be used with in vivo and in vitro evaluation of genotoxicity. The single-cell gel electrophoresis assay detects low levels of DNA damage in a small number of cells. Measurements of genotoxicity are carried out using Comet IV software or manual counting.

### 3.6. BNNTs Biocompatibility In Vitro

BNNTs in biomedical applications have been studied in in vitro assessments using various cell lines to understand the toxicity levels and biocompatibility. Ciofani et al. [82] (in 2008) first reported the toxicity of BNNTs, using SH-SY5Y cells. The BNNTs were functionalised with polyethyleneimine (PEI) and incubated in the cell line for up to 72 h to evaluate the toxicity [82]. The viability tests were carried out using Trypan Blue and MTT assay [82]. The results stated that PEI-coated BNNTs were non-cytotoxic and displayed no negative effects on cell functions [82]. Furthermore, it was mentioned that due to the PEI on the surface of the BNNTs, the cell-uptake mechanisms were not impacted. The outcomes of the study stated that BNNTs did not show any significant toxicity levels, up to 5 µg·mL^−1^ for up to 72 h [82]. Similarly, Raffa et al. [79] demonstrated the toxicity of BNNTs using the same cell lines with different functionalisation material. The study as carried out on PLL-coated BNNTs (PLL-BNNTs). Furthermore, the BNNTs were tested as a nanotool to facilitate cell electropermeabilisation (EP) with extremely low electric fields varying from 40–50 V/cm [79]. The results demonstrated that the presence of BNNTs (facilitated with EP) displayed high cell viability, metabolism, and proliferation. In addition, BNNTs mediated with EP aided in increasing cell permeabilisation with low voltage to allow chemicals or DNA to be introduced into cells for drug delivery and therapeutic treatments [79]. Using the same human neuroblastoma cells, Ciofani et al. [76] explained the cytocompatibility and stability that was attained through BNNTs wrapped with glycol-chitosan (GC-BNNTs). The various concentrations of GC-BNNTs (0, 5, 10, 20, 50 and 100 μg·mL^−1^) were incubated in cells for 48 h and assessed with both MTT and WST-1 assay [76]. The results stated that the BNNTs at higher concentrations showed optimal cytocompatibility with no adverse effects on viability, toxicity, early apoptosis, and ROS. Moreover (and interestingly), the results stated that the MTT assay showed false cytocompatibility findings due to the BNNTs interactions with tetrazolium salts that hindered the results, which showed a viability decrement of 10 µg·mL^−1^. However, a water-soluble assay, namely WST-1, indicated that intrusion did affect the enzymatic reaction, with no decrease in viability at 10 μg·mL^−1^ [76]. Furthermore, the early apoptosis detection and ROS detection performed after 48 h showed no evidence of significant negative effects with cells incubated with different concentrations of BNNTs-GC [76]. Similarly, other studies carried out using the same cell line showed significant cytocompatibility in vitro, up to 100 µg·mL^−1^ [68,73] (See Table 2). 

#### 3.6.1. Human Embryonic Kidney Cells

Chen et al. [81] showed the biocompatibility of BNNTs with HEK cells. The BNNTs were synthesised using a chemical vapour technique (CVD) with a geometry of 20–30 nm diameter and lengths ranging up to 10 mm [81]. The BNNTs were incubated in HEK 293 cells without any functionalisation, up to 100 mg·mL^−1^ [81]. Additionally, the toxicity of BNNTs was compared with CNTs of similar length and diameter. The results indicated that BNNTs showed similar cell growth in control (cells cultured in a medium without nanotubes) [81]. In contrast, CNTs showed a significant decrease in cell growth after 4 days [81]. Furthermore, the apoptosis and necrosis analysis with annexin V-FTIC/propidium staining on cells treated with BNNTs and glycodendrimer coated BNNTs observed that BNNTs directly bind to cell surfaces [81]. This easy method demonstrated that coated BNNTs and uncoated BNNTs revealed comparable dendrimer-bearing galactose deposits that were adept at cooperating with proteins and cells [81].

#### 3.6.2. T98g and Fibroblast Cells 

In another study, the cytocompatibility of BNNTs with yields of 80 and 97% purity was functionalised with PLL (PLL-BNNTs) and was evaluated using T98G cells and human gingival fibroblasts [80]. Furthermore, the functionalised BNNTs were bound with folic acid (FA) to obtain folate conjugated PLL-BNNTs (FA-PLL-BNNTs) [80]. Both the PLL-BNNTs and FA-PLL-BNNTs were covalently identified with carboxyl-derivatised quantum dots for cellular-tracking studies [80]. After 24 h of treatment at 10 µg·mL^−1^ concentrations of both PLL-BNNTs and FA-PLL-BNNTs, the contents displayed complete cytocompatibility of PLL-BNNTs with both cell lines [80]. Furthermore, cell viability assayed using Trypan Blue displayed >95% viability in each case [80]. The MTT assay displayed excellent metabolic activity (80%) for both cells, with no significant difference to the control [80]. 

#### 3.6.3. Human Osteoblasts Cells 

Danti et al. [66] investigated the toxicity of PLL-BNNTs using a different cell line (primary human osteoblasts (HOBs)) and the effects of BNNTs with an ultrasound stimulatory method on cell function and maturation was studied [66]. The MTT viability assay displayed excellent metabolic activity of the treated cells with PLL-BNNTs after 72 h [66]. The early apoptotic and necrotic phenomenal showed no substantial variation in both control and PLL-BNNTs-treated cells after 24 and 72 h [66]. Furthermore, the osteoblast-cell internalising BNNTs that were irradiated with low-frequency ultrasound showed improved in protein concentration with respect to the controls [66]. Lahari et al. [77] addressed the cytotoxicity of the BNNTs-reinforced polylactide-polycaprolactone copolymer (PLC) composites using both HOBs and macrophage cells. The cytotoxicity evaluation was carried out using an LDH assay and showed no adverse effects of the BNNTs-reinforced polymer in both cells [77]. Furthermore, BNNTs-reinforced PLC increased the cell viability rate on composite films. More interestingly, it was observed that fourfold and sevenfold increases occurred in levels of expression of transcription factor Runx2 in composite films [77]. The same group studied the biocompatibility of hybrid composite produced by BNNTs mixed with hydroxyapatite (BNNTs-HA) using HOBs [75]. The results stated an accelerated osteoblast-cell viability and proliferation within the presence of BNNTs [75].

In another study, Fernandez-Yague et al. [54] validated BNNTs functionalised with polydopamine (PD) cytocompatibility using HOBs at concentrations of 1, 10, and 30 μg·mL^−1^. The quantification of cell viability with PD-coated BNNTs-treated cells showed good metabolic activity with a 90% proliferation rate, while uncoated BNNTs showed reduced metabolic activity and proliferation rates compared to control conditions [54]. Moreover, the study stated that PD-coated BNNTs localised to the HOB plasma membrane can be deposited on the cell surface, acting as a protective layer and preventing endocytosis of isolated nanotubes [54]. Furthermore, the PD-coated BNNTs were internalised by cells as individual entities [54]. Another interesting investigation reported by Farshid et al. [47] was on BNNTs-reinforced poly(propylene fumarate) (PPF-BNNTS) in vitro cytotoxicity, using MC3T3 pre-osteoblasts. The cell viability was determined by the resazurin-based Presto Blue^®^ assay. It was reported that 1 μg·mL^−1^ PFF-BNNTs showed 100% cell viability while 100 μg·mL^−1^ showed 99 ± 13% [47]. The results suggested good cell viability, attachment and spreading of MC3T3 cells on all experimental groups [47].

#### 3.6.4. Fibroblast Cells 

Ciofani et al. [70] reported on in vitro biocompatibility and cellular uptake of BNNTs functionalised with amino salts, using fibroblast (NIH/3T3) cells. An aminosliane named APTES was presented as a surface functionalisation agent for the BNNTs. This opened up various interesting perspectives for BNNTs modification using biomolecules [70]. The obtained BNNTs-coated APTES were incubated with NIH/3T3 cells for up to 72 h to study the viability rate of cells [70]. The WST-1 assay showed (after 24 h) excellent metabolic activity and viability of cells treated with 100 µg·mL^−1^ functionalised BNNTs [70]. However, after 72 h a decrement in viability rate was observed—about 16% at both 50 and 100 µg·mL^−1^ concentration of BNNTs [70]. DNA concentration analysis showed similar results as the WST-1 assay with no significant effects after 24 h at higher concentrations. While there were decreases up to 20% at 50 µg·mL^−1^ and 100 µg·mL^−1^ BNNTs concentration after 72 h [70]. The confocal microscopy images of actin-stained cells treated with f-BNNTs displayed no evidence of f-BNNTs in cell nuclei [70]. Consequently, f-BNNTs demonstrated optimal cytocompatibility at higher concentrations, with stable dispersion [70].

Emanet et al. [48] prepared the hydroxylated BNNTs (BNNT-OH)-chitosan scaffold and tested their mechanical strength, swelling behaviour and biodegradability. The results showed that the inclusion of BNNTs-OH into the chitosan scaffold increased the mechanical strength and pore size at optimal for high cellular proliferation and adhesion [48]. The chitosan-BNNT-OH scaffold was also found to be non-toxic to human dermal fibroblast (HDF) cells due to their slow degradation rate. The results were confirmed with DAPI-stained cells proliferated on a chitosan-BNNT-OH scaffold better than the cells on the chitosan-only scaffold [48]. 

In another study, Emanet et al. [55] reported functionalisation of the BNNTs by hydroxylation (h-BNNTs) and carbohydrate modification to increase the cellular uptake and dispersibility of the nanotubes. Glucose, lactose, and starch-modified BNNTs (m-BNNTs); BNNTs and h-BNNTs (5–200 μg·mL^−1^) were incubated (1–3 days) with two cell lines (HDF and A549) to evaluate the cytotoxicity and the genotoxicity [55]. The A549-cell viability declined to 40% and 60%, while the HDF-cell viability reduced to 90% during the second and third days of the incubation with BNNTs and h-BNNTs [55]. In addition, m-BNNTs showed no adverse effects on the viability of HDFs and A549 cells [55]. Meanwhile, the ROS production significantly increased in BNNTs and h-BNNTs-exposed cells up to 70 and 110%, in both cases [55]. In contrast, the ROS production was not significant (20 and 30%) in m-BNNT-exposed cells, with respect to the control cultures [55]. With the comet assay, BNNTs and h-BNNTs-treated cell tail-lengths were approximately 38%, while the m-BNNT-exposed cell tail-lengths were 20% and 30% if compared to the positive control cells, which were exposed to hydrogen peroxide [55]. Hence, the outcomes showed that the increase in ROS levels in the cells was due to DNA damage [55]. Furthermore, the analysis indicated that BNNTs and h-BNNTs were cytotoxic, but m-BNNTs were biocompatible for various biomedical applications without significant damage to healthy cells [55]. 

**Figure 5:**
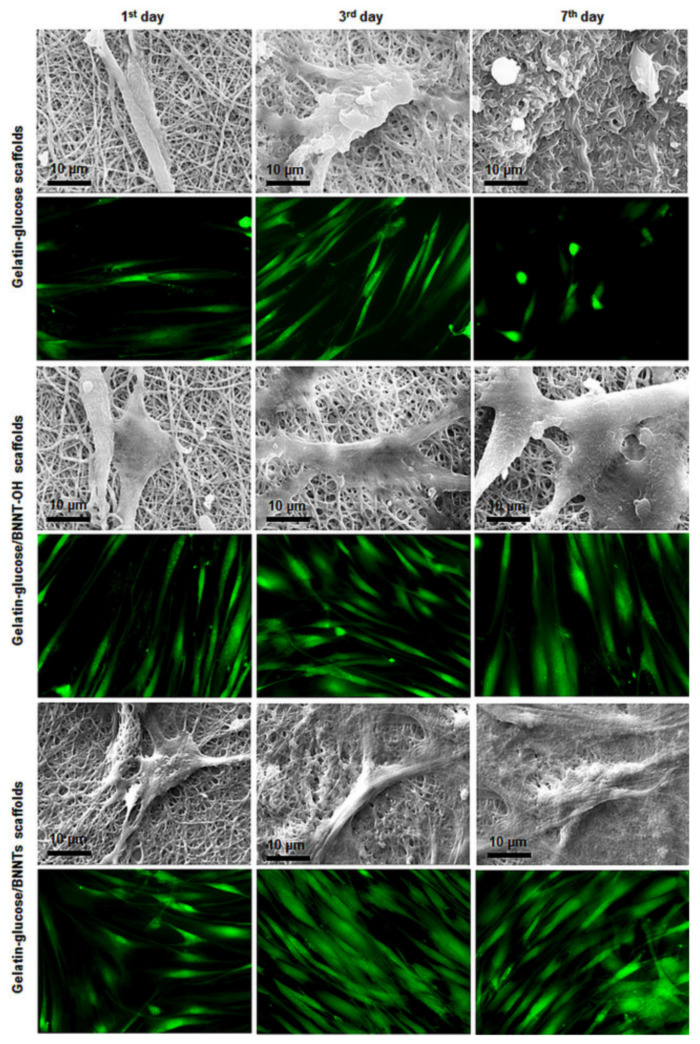
Microscopy images of HDF cells growing onto the BNNTs reinforced gelatine and glucose scaffolds. Reproduced with permission from Ref. [51]. Copyright 2015, Elsevier.

Sen et al. [51] studied the biocompatibility of BNNTs-reinforced gelatine and glucose scaffolds produced using an electrospinning technique. The biocompatibility tests were carried out using HDF cells incubated with BNNTs-gelatine scaffolds after exposure for up to 7 days [51]. The results stated that the scaffolds did not substantially impact the cell viability rate (Figure 5) [51]. In other studies, Diez-Pascual et al. [53] reported polyethylene glycol (PEG)-grafted BNNTs-reinforced poly (propylene fumarate) (PPF) nanocomposite biomaterials for tissue-engineering applications. The cytotoxicity of PPF-PEG-g-BNNT nanocomposites was assessed by culturing with HDF [53]. The viability results of various concentrations of PPF-PEG-g-BNNT (0.0–4.0 wt%) showed negligible toxicity towards HDF cell lines after 24 h [53]. Thus, the covalent grafting of BNNTs with PEG reduced the cytotoxicity towards the cells and likely aid in good dispersion of BNNTs in aqueous media [53]. 

Ferreira et al. [65] investigated the cytocompatibility of BNNTs functionalised with organic hydrophilic agents composed of glucosamine (GA), polyethylene glycol (PEG) 1000 and chitosan (CH). The in vitro studies of f-BNNTs were conducted using lung fibroblast MCR-5 cells [65]. The functionalised BNNTs (0.1–50.0 µg·mL^−1^) treated with cells showed an approximately 90% viability rate after 48 h [65]. At higher concentrations of 100 and 200 µg·mL^−1^ the viability was approximately 50% and 25% in samples of BNNTs-CH and BNNTs-PEG [65], while BNNTs-GA maintained excellent viability (>90%) up to 100 µg·mL^−1^ after 48 h. Furthermore, with BNNTs-GA, the results stated that no significant chromosomal damage, DNA damage, or ROS increase was observed, up to a concentration of 50 µg·mL^−1^ [65]. However, concentrations above 50 µg·mL^−1^ in BNNTs-CH and BNNTs-PEG resulted in significant cell damage and increased cytotoxic activity [65]. 

Degrazia et al. [41] reported the cytotoxicity of a methacrylate-based adhesive containing BNNTs using fibroblast cells in derived dental pulp. The results stated that cell viability of fibroblasts after 72 h was enhanced up to 10% when 0.05 wt% BNNTs were incorporated into methacrylate-based adhesive [41]. Furthermore, it was stated that the cytocompatibility depended on the purity, concentration, and functionalisation of BNNTs [41]. Similar studies have been conducted by Bohns et al. [34] and Barachini et al. [62] to analyse the BNNTs cytotoxicity with fibroblast cells derived from dental pulp. It was reported that BNNTs incubated with cells do not have any significant effect on the viability of cells. 

#### 3.6.5. HeLa Cells 

**Figure 6:**
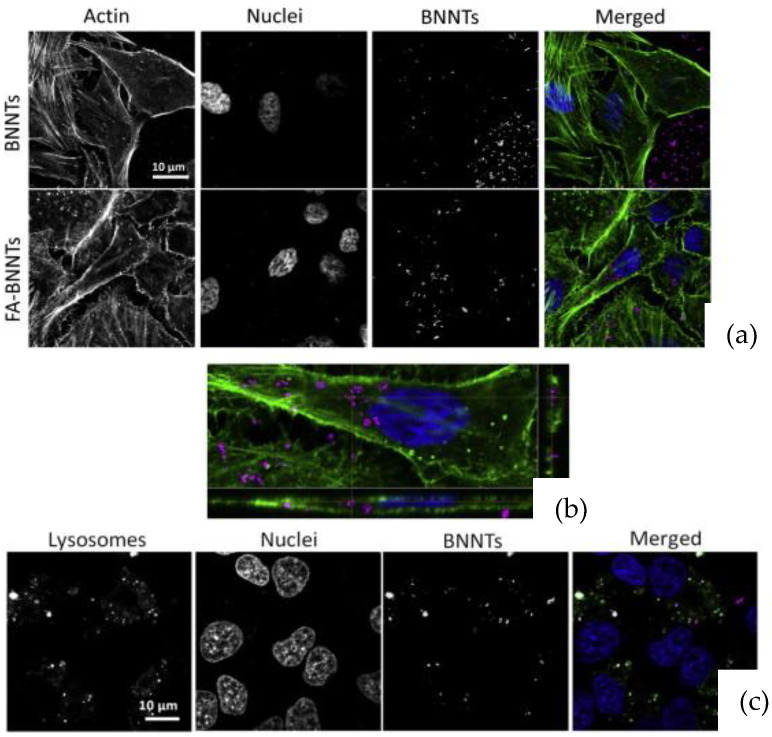
(**a**) Confocal images of HeLa cells treatment with BNNTs and FA-BNNTs; (**b**) FA-BNNTs internalisation by HeLa cells; (**c**) lysosome staining (in green) for FA-BNNT (in pink) co-localisation evaluation. Reproduced with permission from Ref. [58]. Copyright 2015, Elsevier.

Ferreira et al. [43] investigated the cytocompatibility of BNNTs using HeLa cells. Concentrations of 10, 50, 100, and 200 µg·mL^−1^ of BNNTs were incubated with cells for 2 days and viability was assessed using WTS-1 assay [43]. The results showed that the viability rate was more than 80% for all concentrations [43]. Furthermore, a cell-irradiation assay was carried out to promote cell-death signalling in tumour cells, which showed minor toxicity where BNNTs were internalised [43]. Overall, the biological assays showed that the BNNTs had a suitable cell viability and that irradiation with an appropriate flux of thermal neutrons did not cause significant damage in the cells studied [43]. In another study, BNNTs functionalised with folic acid (FA) were incubated with HeLa cells to evaluate the cytocompatibility [58]. The in vitro assays tests indicated that no adverse effects were found on HeLa cells cultured with FA-BNNTs and BNNTs in a concentration range of 0–50 µg·mL^−1^ [58]. Furthermore, the internalisation assessment revealed that BNNTs were located outside the cells, while FA-BNNTs were highly internalised by the cells, indicating an active role in the cell-uptake process (Figure 6) [58].

#### 3.6.6. Human Umbilical Vein Endothelial Cells

Del Turco et al. [67] also studied the effects of GC-BNNTs (0–100 µg·mL^−1^) incubated with HUVECs for 48 and 72 h. It was reported that no adverse effects were identified in cell viability, the cytoskeleton, or DNA damage. Another interesting study used gum Arabic (GA) as a non-covalent functionalisation agent to obtain dispersion and stability of BNNTs [64]. The obtained gum-Arabic-coated BNNTs were cultured in both HUVECs and SH-SY5Y cells, with concentrations of 0–100 µg·mL^−1^ [64]. The viability tests conducted using WST-1 assay for SH-SY5Y and Amido Black assay for HUVEC cells demonstrated that the f-BNNTs viability rate was not statistically different from the control cultures, up to 20 µg·mL^−1^ after 72 h [64]. However, there was a significant decrease in the rate of viability in higher concentrations, after 72 h [64]. The micrograph images showed that cells failed to reach confluence after 72 h in higher concentrations over 20 µg·mL^−1^ [64]. Additionally, the results stated that no adverse toxic effects were displayed (up to 20 µg·mL^−1^) on both cell types in terms of ROS production and apoptosis induction [64]. It was highlighted that BNNTs were suitable for in vitro biomedical applications (up to 20 µg·mL^−1^) with a suitable length and aspect ratio of the nanotubes [64]. 

#### 3.6.7. Human Osteosarcoma Cells 

Marcos da Silva et al. [31] conducted a study using an in vitro assay on BNNTs incorporated with samarium and gadolinium (GdBO_3_-BNNTs) with a human osteosarcoma cell line (SAOS-2) and HDF cells. The samples in a concentration of 10 µg·mL^−1^ showed high biocompatibility both with fibroblasts (92% cell viability) and with SAOS-2 cells (70% cell viability) [31]. A concentration of 50 μg·mL^−1^ indicated low biocompatibility with fibroblasts (50% cell viability) but high biocompatibility with SAOS-2 cells (80% cell viability) [31]. The results suggested that biocompatibility relied on low concentrations and the osteosarcoma cells were more resistant to this material than normal cells [31]. The results showed that the GdBO_3_-BNNTs can be used in scintigraphy radiotracers or as MRI contrast medium, being able to promote the treatment of many types of tumours simultaneously with their diagnosis [31]. Genchi et al. [39] investigated piezoelectric films of BNNTs-reinforced poly(vinylidenedifluoride-trifluoroethylene) (PVDF-TrFE-BNNTs) that were prepared by cast annealing and used SAOS-2 cells to evaluate the cytocompatibility. The percentage of Alizarin Red-stained areas of piezoelectric PVDF-TrFE-BNNTs films was higher with absence or presence of ultrasound. Moreover, the markers were significantly increased in cells cultured on PVDF-TrFE-BNNTs films [39]. 

#### 3.6.8. Mesenchymal Stem Cells 

In another study, Li et al. [52] described the interaction between BNNTs and mesenchymal stem cells (MSCs). The results stated that BNNTs displayed an increase in protein absorption and enhanced the cell proliferation of MSCs that improved the secretion of total protein by MSCs [52]. In addition, BNNTs increased the alkaline phosphate activity as an early marker of osteoblasts and osteocalcin as a late marker of osteogenic differentiation [52]. Overall, it was reported that BNNTs were able to enhance the osteogenesis of MSCs, which showed potential in bone regeneration in orthopaedic applications [52]. Ferreira et al. [60] incubated GA-BNNTs in MSCs to investigate the cytocompatibility. The cell viability and proliferation were not affected, up to 20 μg·mL^−1^ of GA-BNNTs [60]. The cytoskeleton study revealed a significant reorganisation of the forms based on f-actin staining, because of uptake of the GA-BNNTs [60]. Additionally, it was indicated that BNNTs enhanced the differentiation of MSCs into adipocytes (but not into osteocytes) and led to an increase in mRNA level for adipocyte differentiation [60]. 

#### 3.6.9. MDA-MB-231-luc2 Cells 

Sen et al. [46] demonstrated that BNNTs could be functionalised with oligonucleotides (oligo-BNNTs) and chemically hybridised to morpholinos (morpholino/oligo-BNNTs). The hybridised composite used delivery of morpholinos into MDA-MB-231-luc2 cells [46]. The gene-slicing assessment stated that luciferase activity decreased when MDA-MB-231-luc2 cells were treated with morpholino/oligo-BNNTs, which showed gene-slicing efficiency [46]. The WST-1 assay displayed that the scrambled morpholino/oligo-BNNTs, scrambled lipofectamine, morpholino/oligo-BNNTs, and morpholino-lipofectamine did not significantly affect cell viability (88 ± 4% for all) [46]. 

#### 3.6.10. Glioblastoma Cells

Niskanen et al. [50] evaluated the response of cells such as N9 microglia and U25IN glioblastoma to BNNTs coated with glycine and loaded with model drug, curcumin, and fluorescent probes. The length of the BNNTs was approximately 2 µm with the ends opened up due to the sonication process [50]. The cell viability tests uncovered that microglia cell death occurred when exposed to a BNNT concentration over 10 µg·mL^−1^ [50]. However, U25IN cells remained viable when they were exposed to the higher concentration of BNNTs, under the same conditions [50]. In contrast, the viability of U25IN cells was reduced after 24 h of exposure to the higher concentration. Whereas curcumin-g-BNNTs incubated with microglia cells was decreased in the viability at a concentration higher than 50 µg·mL^−1^, the U25IN cell loss was 25.3 ± 6.3% at 50 µg·mL^−1^ [50]. The mitochondrial metabolic activity of cells indicated that drugs such as curcumin can be effectively incorporated into BNNTs, internalised by tumour cells, and can release therapeutic effects [50]. The curcumin-loaded BNNTs reduced the inflammation from microglia cells stimulated with LPS. However, curcumin-g-BNNTs showed reduced metabolic impairment caused by BNNTs devoid of curcumin [50].

#### 3.6.11. Vero, Chang Liver, MCF7, and A549 Cells 

Nitya et al. [63] used BNNTs functionalised with four different surfactants such as Pluronic-P123, PEI, Pluronic-F127, and ammonium oleate (A.O.) to investigate their antibacterial properties and conduct cytotoxic studies. The pristine BNNTs and surfactant-coated BNNTs were evaluated to interpret their antibacterial activity and cytotoxicity levels in various cells such as Vero, Chang liver, MCF7, and A549 cells [63]. The toxicity levels were analysed using an MTT assay. The results stated that the F127-coated BNNTs and pristine BNNTs showed good viability rates in all cell lines, up to 250 µg·mL^−1^ [63]. P123 and A.O-coated BNNTs showed no adverse effects, up to 125 µg·mL^−1^ [63]. However, PEI functionalised BNNTs showed significant toxicity levels on Vero and Chang liver cells at lower concentrations [63]. In addition, DNA fragmentation of F127-coated BNNTs indicated the apoptotic pathway of cell death in cancer cells [63]. 

#### 3.6.12. Other Types of Cells 

Rocca et al. [49] investigated pectin-coated BNNTs (P-BNNTs) incubated with RAW-264.7 macrophages to evaluate in vitro cyto- and immune-compatibility. The WST-1 assay results demonstrated that cell metabolism was not altered by P-BNNTs treatment at all the considered concentrations with respect to the control cultures [49]. The proliferation rate was assessed using Quant-iT™ PicoGreen^®^ ds-DNA assay, which showed no differences in terms of DNA concentration in the treated samples, compared to the control [49]. Flow-cytometry measurements of necrotic/apoptotic phenomena finally exhibited that an acute treatment with P-BNNTs, up to 50 μg·mL^−1^, did not cause a statistically significant growth of necrotic, early apoptotic, or late apoptotic cells in comparison to a control culture [49]. Furthermore, P-BNNTs did not stimulate inflammation responses, both at protein and gene levels [49]. Poudel et al. [38] analysed the cellular response to piezoelectric materials composed of PVDF-TrFE-BNNTs and evaluated cytocompatibility using human-tendon-derived cells. The cell-proliferation assays confirmed that cells cultured on PVDF-TrFE/BNNT nanocomposites demonstrated enhanced proliferation for up to 10 days in culture relative to pure PVDF-TrFE films [38]. 

Pasquale et al. [30] investigated the BNNTs doped with doxorubicin (Dox) and coated with cell membranes (CM) derived from glioblastoma multiforme (GBM-a brain-cancer cell type) cells (Dox-CM-BNNTs) that are able to kill GBM cells in vitro while leaving healthy brain cells unaffected. The anti-cancer properties of Dox-CM-BNNTs at various concentrations such as 25, 50, 100, and 200 μg·mL^−1^ were examined on U87 MG glioblastoma cells and related to the cytotoxicity of CM-BNNTs and mPEG-DSPE-BNNTs [30]. The results showed that cell viability decreased in both Dox-CM-BNNTs and free Dox after 24 and 72 h in all concentrations [30]. However, the CM-BNNTs and mPEG-DSPE-BNNTs did not show any significant effects on the cells [30]. The reports highlighted that 100 μg·mL^−1^ Dox-CM-BNNTs displayed a significant cell-death rate and resulted in anti-cancer effects [30]. 

**Figure 7:**
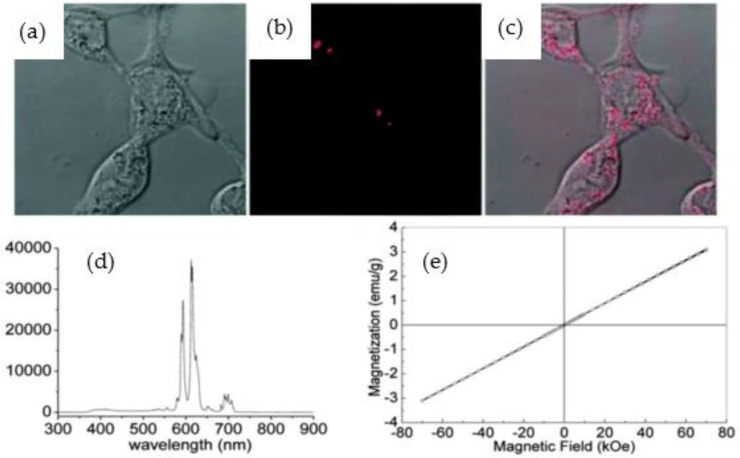
(**a**) Fluorescence image of Dox loading BNNTs@NaGdF_4_: Eu; (**b**) LNCaP prostate cancer cells fluorescence image; (**c**) Overlapped fluorescence image in red colour emissions of composites uptake by cancer cells; (**d**) Photoluminescence emission spectrum loading BNNTs@NaGdF_4_:Eu; (**e**) Function of magnetisation at room temperature for BNNTs@NaGdF_4_:Eu composites. Reproduced with permission from Ref. [61]. Copyrights 2014, Royal Society of Chemistry.

Li et al. [61] examined the utility of BNNTs@europium-doped sodium gadolinium fluoride (BNNTs@NaGdF_4_:Eu) for fluorescence imaging and magnetic targeting, especially for cancer therapy (Figure 7). BNNTs@NaGdF_4_:Eu was incubated with human LNcaP prostate-cancer cells to evaluate the influence of a permanent magnetic field and in vitro cell uptake [61]. The fluorescence intensity results indicated that for mediums containing 20 μg·mL^−1^ of BNNTs@NaGdF_4_:Eu, a significant cell uptake of 404 ± 30 was observed in the presence of a magnetic field and in the absence of a magnetic field it was 315 ± 18, while for pure cells in the presence of a magnetic field it was 230 ± 3 [61]. Furthermore, the cancer-cell viability in the absence and presence of a magnetic field was measured through 30% of Dox loading BNNTs@NaGdF_4_:Eu [61]. The viability rate of human LNcaP prostate cancer decreased after exposure to dox-BNNTs@NaGdF_4_:Eu in the presence of a magnetic field [61]. In contrast, in the absence of a magnetic field, the value was higher than it was in the presence of a magnetic field. Moreover, the multifunctional BNNTs composites showed potential in enhancement of chemotherapy with efficient use of magnetic fields [61]. 

Ferreira et al. [32] validated the tumour-homing peptide CREKA functionalised BNNTs (BNNTs-CREKA) effects on 4T1 tumour cells. The in vivo analysis observed that a significant amount of BNNTs-CREKA piled up at the tumour to target the cells [32]. Furthermore, biodistribution studies were conducted in mice after injecting radioactive 99m-BNNTs-CREKA [32]. The radioactivity biodistribution was assessed by an automatic scintillation counter in liver, spleen, kidneys, stomach, thyroid, heart, intestines, tumour, and muscle after exposure for up to 8 h with organs removed from mice [32]. It was observed that the tumour uptake was higher compared to non-targeted tissues such as muscle, intestines, heart, and thyroid, while the uptake was higher in liver, spleen, and kidney due the macrophages present in these organs after exposure [32]. Similarly, Nakamura et al. [59] synthesised BNNTs functionalised with DSPE-PEG2000 to investigate antitumour outcomes om B16 melanoma cells [59]. The results indicated that BNNTs-DSPE-PEG2000 showed higher antitumour effects in B16 cells [59].

In another study, Danti et al. [56] presented an interesting analysis on BNNT functionalised muscle-cell microfibre-mesh scaffolds acquired through a tissue-engineering three-dimensional (3D) platform to study a wireless stimulation system for electrically responsive cells and tissues [56]. The scaffolds were seeded with C2C12 myoblast cells under low ultrasound (US) irradiation [56]. The results stated that the cells’ interaction with BNNTs increased gene (Cx43) expression in 3D samples. Additionally, the higher protein levels of Cx43 and myosin were detected in the 3D scaffold model. The findings indicated that there was potential for developing appropriate in vitro platforms for biological modelling [56].

#### 3.6.13. In Vitro Studies Stated BNNTs Are Cytotoxic 

The above-mentioned studies were generally in favour of BNNTs biocompatibility. However, there are debatable results about their toxicity in the literature. Horvath et al. [74] highlighted the toxicity of pure BNNTs, which were dispersed with Tween 80 on various cell lines, such as lung epithelium cells, mouse macrophage cells, mouse embryonic fibroblasts, and HEK 293, with a maximum BNNT concentration of 20 µg·mL^−1^ [74]. Various viability assessments were performed and cytotoxic effects at a minimal dose of BNNTs incubated with cells was noticed [74]. The study highlighted the size and aspect ratio and absence of biomolecules that lead to higher toxicity levels in cell cultures [74]. Augustine et al. [37] investigated a novel atomic force microscopy (AFM)-based cardiomyocyte assay that reliably assesses the cytotoxicity of BNNTs. High-energy probe sonication was used to modify and control the length of BNNTs [37]. Cytotoxicity studies using the novel cardiomyocyte AFM model agreed with traditional colorimetric cell metabolic assays, both revealing a correlation between tube length and cytotoxicity with longer tubes having higher cytotoxicity [37]. In addition to the size-dependent cytotoxicity, it was found that BNNTs exhibited concentration- and cell-line-dependent cytotoxic effects [37]. Çal et al. [35] demonstrated the cytotoxicity and DNA damage effects of BNNTs and curcumin using HeLa cells, CD34+ cells, and V79 cells. The MTT and Comet assay indicated that curcumin and BNNTs-curcumin were cytotoxic in all the concentrations [35]. Furthermore, the increase in ROS caused DNA damage to cells treated with BNNTs. The results stated that curcumin and BNNTs-curcumin concentration groups were similar, which may be a sign of the BNNTs inertness [35].

Overall, the results of BNNTs in in vitro toxicology depend on the purity, functionalisation, and geometrical dimensions of BNNTs. Taken together, all the literature on in vitro assessment of BNNTs showed good biocompatibility. The biocompatibility data for in vitro conditions reported thus far (on various cell lines) stated that BNNTs are potential nanomaterials for nanovectors, therapeutic, and biomedical applications although further in vivo and clinical analysis is needed.

### 3.7. BNNTs Biocompatibility In Vivo

The biocompatibility of BNNTs should be further assessed to identify the toxicity levels to tissue at the organism level through in vivo studies. The in vivo studies have been conducted using BNNTs, either pristine or functionalised, and various dispersion agents injected/fed into the animals. The animals were generally injected or fed with various dosages of BNNTs to investigate the toxicity levels or unusual inflammation reactions in their airways or blood or distribution of BNNTs in the organisms. 

To date, in vivo assessment of BNNTs is very limited. An initial pilot study was carried out by Ciofani et al. [72] which was performed with BNNT-GC (1 mg·mL^−1^) injected into the marginal ear veins of five rabbits [72]. The blood tests were performed up to 72 h after injection and compared with plain GC (1 mg·mL^−1^) solution [72]. The results stated that there was no alteration of the basic hematic parameters that could subtend the functional impairment of blood, liver, or kidneys [72]. No acute toxicity was observed after BNNTs-GC was injected into rabbits over the period [72]. In another investigation by Ciofani et al. [69], a higher dose of up to 10 mg·kg^−1^ of GC-BNNTs was injected into the rabbits. Additionally, an assessment was performed after injecting 5 mg·kg^−1^ of GC-BNNTs once per day for three days [69]. The blood-analysis report showed no evidence of negative effects on blood or liver and there was no kidney impairment [69]. Furthermore, plasma pharmacokinetic studies indicated no significant temporary accumulation of BNNTs in tissues that could act as reversible reservoirs [69]. Collectively, these data suggest a relatively high clearance of BNNTs from the blood and a quick distribution in the organism and/or excretion [69].

**Figure 8:**
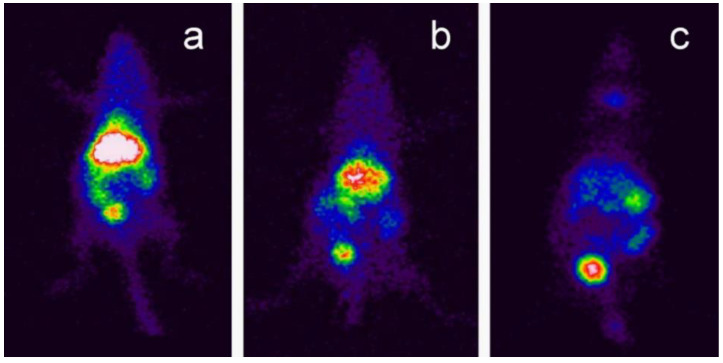
Scintigraphic image of radiographic GC-BNNTs biodistribution in mice; (**a**–**c**) show the images after injecting at time intervals of 30 min, 1, and 4 h, respectively. Reproduced with permission from Ref. [71]. Copyrights 2012, Elsevier.

Soares et al. [71] demonstrated BNNTs functionalised with GC and radiolabelled with ^99m^Tc and injected into Swiss mice tails in order to evaluate their biodistribution. The results showed that, after 24 h, GC-BNNTs had accumulated in the liver, spleen, and gut, and had been eliminated via renal excretion (Figure 8) [71]. 

Salvetti et al. [57] reported the effects of BNNTs on stem cells and tissue regeneration in planarians. BNNTs coated with GA were injected into planarians at concentrations of 100 or 200 µg·g^−1^ for 15 days [57]. It was stated that GA-BNNTs were internalised by intestinal cells within 1 day after injection and did not induce any DNA damage in animals [57]. However, the study failed to detect the difference in expression levels of molecular markers specific to stem cells and stem-cell progenies that indicate BNNTs effects in stem cells [57]. The study found no adverse effects on neoblasts, which are essential for tissue regeneration [57]. Furthermore, the analysis stated that GA-BNNTs did not show any effects in the morphogenetic process [57]. 

Demir et al. [40] studied the antioxidant/antigenotoxic properties of BNNTs using drosophila melanogaster. The analysis stated that non-relevant genotoxic effects were observed in the wing-spot assay or in the Comet test [40]. Furthermore, it was observed that BNNTs significantly reduced the genotoxic effect of potassium dichromate (PDC) and the intracellular levels of ROS, which indicates the non-toxic effects of BNNTs [40]. 

**Figure 9:**
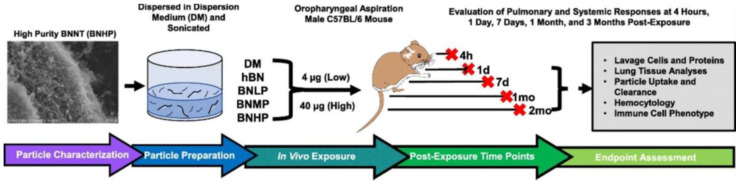
Overview of the BNNTs toxicity investigation following pulmonary exposure in mice. Reproduced with permission from Ref. [29]. Copyrights 2020, Elsevier.

In contrast to the above studies, Xin et al. [29] reported the toxicity of BNNTs after being exposed to lung cells, using an in vivo time-course model. The in vivo studies were performed on male C57BL/6J mice with BNNTs (~50% purity) at 200 µm length and 5 nm width (Figure 9) [29]. BNNTs at 4 and 40 µg concentrations were exposed for 4 h, 1, 4, and 7 d, 1 and 2 months to mice (lungs) to measure and evaluate pulmonary and extrapulmonary toxicity [29]. Bronchoalveolar lavage (BAL) was utilised on the BNNTs-exposed mice to collect fluid and BAL cells to demonstrate the toxicity [29]. The results indicated that high doses of BNNTs considerably enhanced the lactate dehydrogenase activity (LDH) from 4 h to 7 d post exposure, compared to low dose and control groups [29]. However, the low dose of BNNTs did not show any significant lung injury as indicated by LDH activity throughout the time points [29]. Furthermore, the cells displayed a minimal level of inflammation in the high-dose group with resolution over time and no fibrosis [29]. In addition, the lung-clearance analysis observed that ~50% of the BNNTs cleared over the time period [29]. The lung gene expression of Cxcl2, Ccl2, Il6, Ccl22, Ccl11 and Spp1 was considerably increased, 4 h and 1 d after exposure at 40 µg [29]. However, the inflammation and acute-phase gene expression decreased over the times [29]. Interestingly, 4 µg BNNTs did not show any unfavourable effects in the toxicity results, post exposure [29]. Thus, it was determined that high doses of BNNTs showed acute pulmonary inflammation and injury after 7 days of exposure [29]. 

Similarly, Kodali et al. [45] reported acute toxicity both in vitro and in vivo using commercial-grade BNNTs, which are composed of∼50–60% BNNTs and∼40–50% impurities of boron and hexagonal boron nitride. The studies were conducted both in vitro (using THP-1 and NLRP-3 cells) and by injecting 40 µg of BNNTs into male mice [45]. The in vitro studies stated that the BNNTs exhibited dose-dependent acute toxicity and oxidate stress [45]. The results were further confirmed with in vivo tests following a BNNTs exposure, with an increase in bronchoalveolar lavage levels of LDH, a pulmonary polymorphonuclear cell influx, loss in mitochondrial membrane potential, and higher accumulation levels of 4-hydroxynonenal [45]. Additionally, cytokine analysis displayed acute inflammation following the exposure of BNNTs to both cells and in vivo [45].

To summarise, based on the reports available, it was evident that the as-synthesised BNNTs with impurities cause acute toxicity in vivo as well as in vitro. Furthermore, BNNTs functionalised with various materials showed cytocompatibility up to a maximum concentration of 100 µg·mL^−1^. However, further in-depth analysis of BNNTs in various in vivo aspects could give a better understanding of BNNT biocompatibility.

## 4. Biomedical and Tissue-Engineering Applications

Due to their interesting physiochemical properties, BNNTs have been gaining significant attention from researchers and industries. In biomedical and tissue-engineering applications, when BNNTs are functionalised with various organic and inorganic materials, non-toxicity is reported, up to dosage levels of 100 µg·mL^−1^. Thus far, some of the studies have suggested applications of BNNTs in cancer-tumour treatment [32,61,80], drug carries or drug delivery [30], radioisotope accumulation of tumours [31], MRI contrast agents [68,73], reinforcement for biomaterials to produce tissue scaffolds [53,56], orthopaedic procedures [75,77], dental procedures [41,62], bioimaging [158], and bioprinting [24,25]. Not only are there biocompatibility properties but there are also amazing piezoelectrical properties, leading some to propose BNNTs as nanotransducers for the electrical stimulation of cells [39,56].

### 4.1. Boron Neutron Capture Therapy (BNCT)

Cancer is one of the significant causes of death in humans worldwide. Researchers have focused on finding a novel material for targeting the tumour cells with radiation therapy and chemotherapy. In this regard, BNNTs have been investigated as potential material to target cancer cells. For instance, Li et al. [28] demonstrated the Auristatin-PE-coated BNNTs as a drug delivery system to act against the liver cancer cells. The outcomes stated that the PE-BNNTs killed tumour cells and showed promise for treating liver cancer. Furthermore, Li et al. [61] stated that BNNTs@NaGdF_4_:Eu was a possible material with the ability to use in chemotherapy drug delivery systems in the presence of a magnetic field. Similarly, Nakamura et al. [59] stated that functionalised BNNTs displayed a higher accumulation of tumour cells with a combination of thermal neutron irradiation on BNCT. 

### 4.2. Nanovectors

Recent evaluations on the interaction between the BNNTs with living cells confirmed BNNTs as promising nanovectors for various applications in biomedicine; for instance, PEI-coated BNNTs combined with fluorescent markers demonstrated as nanovectors for cell therapy by tracking their uptake by SHY-SY5Y cell lines [83]. Similarly, PLL-BNNTs were demonstrated as nanovectors that can enter the cells when exposed to the electroporation to a 40–60 V·cm^−1^ electric field. 

### 4.3. Tissue Engineering

Recent studies demonstrated that BNNTs combined with various polymeric materials can be used as scaffolds for tissue engineering applications. The scaffolds can be developed using various tissue engineering techniques such as electrospinning or additive manufacturing. For instance, BNNTs combined with co-polymer PLC films were demonstrated as scaffolds for orthopaedic applications with excellent mechanical and biocompatible properties [77]. Similarly, BNNTs combined with resin-based dental sealants showed potential materials in therapeutic procedures of dental hard tissues [34]. In another study, Kakarla et al. demonstrated that BNNTs reinforced gelatine and alginate as a hydrogel to produce hydrogel scaffolds for tissue engineering applications [24,25]. 

Most of the biocompatibility analysis findings with BNNTs’ interaction with different live cells have provided more profound insights. Taken all together, the studies have reported that BNNTs are a promising nanomaterial for biomedical and tissue-engineering applications. 

## 5. Summary and Outlook 

The selection of suitable biomaterials for applications in biomedical research is often associated with the materials’ interactions with living matter. Therefore, the biosafety and biological properties of the material must be considered. Regarding the use of BNNTs, the exploitation of biological properties and interactions with various cell lines and living matter is still at the entry level. A number of challenges still need to be addressed before BNNTs are validated for clinical applications. One of the major issues is BNNTs insolubility in aqueous media. Various organic and inorganic materials have been reported for the functionalisation of BNNTs to obtain stabilised and dispersible BNNTs in aqueous media. However, only a few biomolecules and biocompatible materials have been explored in functionalised BNNTs. Therefore, a wide range of BNNTs (functionalised with various biomaterials or biomolecules) need to be evaluated in vivo and in vitro to understand the toxicity levels. Another major aspect is to find BNNTs toxicity in the living body. Thus far, only a few studies have reported on living-body experiments with commercially available BNNTs and functionalised BNNTs. The results are contradictory, as the studies are limited. Hence, further studies focused on BNNTs in various living organisms and the effects on tissues and organs could help to increase the potential biomedical application of BNNTs. 

For instance, as an innovative nanomaterial, BNNTs show a great range of promising results in cancer treatments, especially in boron-neutron cancer treatment. Additionally, BNNTs aid in increasing bright fields in MRI and creating piezoelectrical material to stimulate cells—these show potential for biomedical applications. However, it was reported that BNNTs with impurities (composed during synthesis or catalyst) displayed acute toxicity under in vitro and in vivo conditions. Thus, it is mandatory to address the influence on cytotoxicity of the impurities or catalysts used in the synthesis of BNNTs. 

In summary, the data as a whole suggested that BNNTs with various functionalised materials were not cytotoxic in concentrations up to 100 µg·mL^−1^. Furthermore, the results confirmed the high potential of BNNTs in various biomedical and tissue-engineering applications. Simultaneously, it was reported that BNNTs are able to address cell behaviour and probe morphological and functional signatures of tumours. However, the candidacy of BNNTs as being optimal for an impressive variety of applications in the biomedical domain needs to be explored more. The biocompatibility of BNNTs under in vivo conditions needs to be assessed further to address biosafety in living organisms. This could pave the way to significant progress in pharmacology, nanomedicine, and even in clinical research.

## Figures and Tables

**Table 1:** List of keywords to identify the articles in Scopus and WOS.

Keywords
“Boron nitride nanotubes” or “bnnts” and “toxicity” or “in vivo” or “in vitro” or “tissue engineering” or “biomedical”) and (limit—to (doctype, “ar”)) and (limit—to (srctype, “j”)) and (limit—to (language, “English”)) and (limit—to (pub stage,” final”)
“Boron nitride nanotubes” (topic) and “biomedical” (topic) and review articles or proceedings papers or book chapters or early access (exclude—document types) and articles (document types) and English (languages)
“Boron nitride nanotubes” (topic) and “tissue engineering” (topic) and review articles or proceedings papers or book chapters or early access (exclude—document types) and articles (document types) and English (languages)
“Boron nitride nanotubes” (topic) and “toxicity” (topic) and review articles or proceedings papers or book chapters or early access (exclude—document types) and articles (document types) and English (languages)
“Boron nitride nanotubes” (topic) and “in vivo” (topic) and review articles or proceedings papers or book chapters or early access (exclude—document types) and articles (document types) and English (languages)
“Boron nitride nanotubes” (topic) and “in vitro” (topic) and review articles or proceedings papers or book chapters or early access (exclude—document types) and articles (document types) and English (languages)

**Table 2:** Summarised correlation between BNNTs geometry, functionalisation, dosage, and time of exposure on various cells and their outcomes.

Authors and References	Synthesis/Source of BNNTs	Geometrical Dimensions of BNNTs	Functionalisation/Composition of BNNTs	Dosage and Time of Exposure	Animal Model/Cell Line	Physiochemical Characterisation	Biocompatibility and Toxicity Assays	Outcomes
Kakarla et al. [24,25]	Co-precipitation and annealing	Diameter: 70 to 130 nm	Hydroxyl-BNNTs (BNNTs-OH)/BNNTs reinforced alginate and gelatin/BNNTs reinforced alginate hydrogel scaffolds	0.05 to 0.1 w·v^−1^%; up to 72 h	HEK 293T	Scanning electron microscopy (SEM), transmission electron microscopy (TEM), Fourier transform spectroscopy (FTIR), mechanical, thermogravimetric analysis (TGA)	Viability: Trypan blue and Ready Probes™ Cell Viability Imaging Kit (blue/green)	Good printability, mechanical strength, and thermal stability with the addition of BNNTs. Minimal toxicity at higher concentrations of BNNTs.
Evariste et al. [26]	Commercial BNNTs (B and N > 99.9%)	Diameter: 2 to 14 nm	–	0, 0.1, 1, and 10 mg·L^−1^; up to 24 h. Larvae were fed twice daily with BNNTs ground aquarium fish food	*Xenopus laevis*	SEM, TEM, TGA, XRD, and Raman spectroscopy	Micronucleus test, cell cycle analysis, analysis of sequences from gut microbiota survey	The specific surface area of BNNTs was 163 m^2^·g^−1^. Micrographs displayed 2 to 10 walls of nanotubes with a mean outer diameter of 6 ± 2.6 nm. BNNTs possessed minor threat to amphibians.
Li et al. [27]	Solid-state reaction		Folate-conjugated BNNTs and coated with auristatin-phenethylamine (PE) (BNNTs-FA@PE)	0–100 μg·mL^−1^	Hep G2 and L02	TEM, FTIR, ultraviolet-visible (UV-vis) absorption spectroscopy, X-ray photoelectron spectroscopy (XPS), size distribution and zeta potential	CCK-8 assay, cellular uptake, actin staining, in vitro anticancer effects, Annexin V-FITC/ propidium iodide (PI), mitochondrial membrane potential, Western blot analysis, detection of Caspase 3/7 activity	The morphology showed bamboo-like shaped nanotubes with diameter of ≈90 nm. BNNTs displayed photoluminescence emission bands at 419, 489, and 594 nm. FTIR analysis displayed BNNTs-FA and BNNTs-FA@PE had absorption bands at 2937–2829 cm^−1^ and 1250–950 cm^−1^ related to the methylene bands of PE molecules. No toxicity in both cell lines and significant increase in metabolic and cellular uptake.
Li et al. [28]	Solid-state reaction		PE-loaded BNNTs	0–100 μg·mL^−1^	HeP G2 Cells	SEM, TEM, Z-potential, FTIR, UV–vis, XPS	Intracellular uptake, lysosomal staining, actin staining, cell viability, flow cytometry, western blot, Capase-3/7 activity	The morphology images displayed BNNTs bamboo-like structures with good dispersive behaviour. Furthermore, BNNTs showed strong emission bands related to B-N and excellent PL properties in the visible light range. The in vivo analysis displayed good internalisation and stimulated cell apoptosis of BNNTs-PE.
Xin et al. [29]	Commercial BNNTs that contain 50% BNNTs with 5 nm wide and 200 μm long	Length: 200 µm; diameter: 5 nm	–	4 or 40 μg; 4 h, 1–7 days, 1–2 months. The mice were fed with BNNT mixed in dispersion media through an oropharyngeal aspiration.	Male C57BL/6 J mice	SEM, TEM, electron paramagnetic response spectra	Lung lavage, BAL cell differentiation, lactic dehydrogenase activity (LDH), BAL fluid protein analysis, lymphocyte phenotypic quantification, mediastinal lymph node and spleen analysis, white blood cell differentiation, histopathology, macrophage uptake, pulmonary clearance, RNA isolation and gene expression	The micrographic analysis of BNNTs showed an ideal length of nanotubes. The specific surface area of BNNTs was 182.6 ± 2.4 m^2^·g^−1^ with the density of 0.03 g·cm^−3^. Only a higher dosage of BNNTs caused the inflammation and a lower dose did not show any effects in the lung.
Lee et al. [21]	Commercial BNNTs	–	Purified BNNTs	0–100 µg	CHO-K1 and 3T3-L1	SEM, XRD, dispersion stability	Cell viability, drug delivery	The SEM and dispersion stability analysis confirmed the nanotubes in tubular structures with stable dispersion in aqueous media. The XRD analysis observed the hexagonal lattice of B−N in BNNTs. Purified BNNTs showed lower cytotoxic at a higher dosage and efficiently carried the drugs than as synthesised BNNTs
Pasquale et al. [30]		–	BNNTs loaded with dox and coated with cell membranes (CM) (Dox-CM-BNNTs)	25, 50, 100, and 200 μg·mL^−1^; up to 72 h	U87	TEM, FTIR, size distribution, zeta potential, TGA, dynamic light scattering (DLS), bicinchoninic acid assay	Cell uptake mechanism, cell viability	The morphology of BNNTs coated with CM was not precise due to low thickness. The FTIR confirmed that BNNTs coated with CM with presence of peaks related to amino acids of CM proteins. The TGA analysis indicated that the total weight loss of CM-BNNTs was 20%. DLS analysis indicated that negative Z-potential related to stable colloidal solution. Dox-CM-BNNTs and free drug were able to substantially decline the cell viability compared to non-treated controls and BNNT controls.
Marcos da Silva et al. [31]	Chemical vapor deposition (CVD)	–	BNNTs doped in situ with samarium (Sm) and gadolinium (Gd) (SmBO_3_-BNNTs and GdBO_3_-BNNTs)	10 and 50 μg·mL^−1^; up to 24 h	HDF and Sarcoma osteogenic (SAOS-2)	XPS, FTIR, SEM, TEM, X-ray fluorescence spectroscopy (XRF), electron energy loss spectroscopy (EELS), vibrational sample magnetometry (VSM), neutron activation	MTT assay, Calcein/Hoechst assay	SEM and TEM images confirmed that the BNNTs were successfully modified with Sm and Gd with uniform distribution on their surfaces. The XPS and EELS analysis further confirmed the presence of Sm and Gd in the BNNTs. In addition, VSM analysis stated that coated BNNTs exhibited magnetic properties. 50 μg·mL^−1^ of GdBO_3_-BNNTs suggested low biocompatibility with fibroblasts (50% of cell viability), but high biocompatibility with SAOS-2 cells (80% of cell viability).
Ferreira et al. [32]	CVD	Diameter: 30 nm; length: 1 µm	BNNT with the CREKA peptide/99mTc-BNNT-CREKA	100 µL; 1, 4 and 8 h	4T1 tumour cells	SEM, TEM, TGA, zeta potential, FTIR	Biodistribution histopathological and blood clearance analysis; fluorescence microscopy cell images	The SEM and TEM micrographs revealed several nanotubes with ≈10 nm outer wall thickness. BNNTs and coated BNNTs showed good thermal stability. FTIR analysis showed B-N stretching vibrations and additional C—H, O—H and O—C bands in coated BNNTs. BNNTs-CREKA as an effective material for targeting the primary tumour tissues and metastatic tumour sites.
Ferreira et la. [33]	Commercial BNNTs		BNNTs incorporated with alkyl trimethyl ammonium bromide (ATAB)	0 to 0.2 wt%; up to 72 h	HaCaT	FTIR (degree of conversion (DC) analysis), microhardness, contact angle, mineral deposition	Cytotoxicity assay and antibacterial assay	No DC was noted in the samples. The contact angle was higher for functionalised BNNTs. The minerals deposition analysis was displayed higher peak intensities in BNNTs-ATAB. No significant cell viability reduction was observed in the BNNTs-ATAB compared with control groups (90%).
Bohns [34]	Commercial BNNTs	Length: 200 µm	BNNTs reinforced resin-based dental sealants (RBSs)	0.1 and 0.2 wt%	Pulp fibroblasts and human keratinocytes	FTIR, tensile strength, contact angle, surface roughness, colour assessment, Mineral deposition	Sulforhodamine B (SRB) cytotoxicity assay	No evidence of DC in the BNNTs-RBSs. The additions of BNNTs to RBSs did not show a significant difference in tensile strength from RBSs. The contact angle values were adequate even though the incorporation of nanotubes. Lower surface energy was noticed for BNNTs comprising RBSs. BNNTs at 0.1 and 0.2 wt% in RBSs did not show any cytotoxicity effects.
Çal [35]	Commercial BNNTs	Diameter:5 nm	BNNTs incorporated with curcumin	10–300 μg·mL^−1^; up to 24 h	HeLa, V79 and CD34^+^	TEM, zeta potential	MTT assay, comet assay	The TEM images showed the BNNTs with micrometres length and Z-potential with positive z signals for curcumin in the BNNTs. BNNTs and BNNTs-curcumin showed minimal toxicity in all cell lines
Ricotti et al. [36]	Annealing	–	Glycol-chitosan (GC)-BNNTs	10 μg·mL^−1^; for 24 h	HDF and C2C12	Focused ion beam (FIB), ICP-MS, EELS	Quantitative real-time polymerase chain reaction (qRT-PCR), cytokine measurements, calcium transients imaging	FIB images revealed evenly dispersed GC-BNNTs in cell culture medium. The ICP-MS showed highest content of boron in cells treated with GC-BNNTs. EEL spectrum confirmed the presence of GC-BNNTs in sections of C2C12 cells. BNNTs were internalised on the top layer of cells and localised inside C2C12 cells, while no particles were internalised by the HDF cells. In addition, BNNTs stimulate cell differentiation at both gene and protein levels.
Augustine et al. [37]	Thermal plasma	–	–	5 to 10 mg of BNNT in 20 mL glass scintillation vial	NB4, HepG2, U87, and A549	AFM, and probe sonication	WST-8, MTT and monitoring beating behaviour of cardiomyocytes	The AFM analysis of BNNTs displayed that the tubes were ≈300 to 500 nm in length with 2 to 3 nm in height. While after probe sonication, the length of nanotubes decreased to 191.9 ± 5.2 nm. BNNTs displayed cytotoxic to the cells measured through AFM-based cardiomyocyte assay.
Poudel et al. [38]	Commercial BNNTs	20–30 µm thickness	Polyvinylidene fluoride (PVDF) and the trifluoroethylene (TrFE) reinforced with BNNTs (PVDF-TrFE-BNNTs)	10 days	Human tendon derived cells	DSC, FTIR, differential scanning calorimetry (DSC), tensile analysis, electrical poling, quasi-static measurement of piezoelectric coefficient	Fibronectin functionalisation, live/dead assay, cell proliferation assay	Addition of BNNTs was evident in enhancing mechanical properties, melting and crystallisation temperatures, and crystallinity. PVDF-TrFE-BNNTs nanocomposite displayed enhanced cell attachment and proliferation compared to pure PVDF-TrFE
Genchi et al. [39]	Pressurised vapor/condenser (PVC)	–	PVDF-TrFE-BNNTs	–	Saos-2	SEM, TEM, AFM, piezo response, piezoelectric transduction, numerical simulation	Cell differentiation, cell stimulation, alizarin red and collagen staining, quantitative real-time reverse transcriptase polymerase chain reaction	The micrographs of BNNTs revealed bundles of nanotube ranging up to µm in length. AFM topographic maps of the PVDF-TrFE-BNNTs showed ~30 nm of mean surface roughness with good piezo electric properties. The piezoelectric films of PVDF-TrFE-BNNTs indicated increased cell differentiation.
Demir et al. [40]	Commercial BNNTs	Average diameter 239.7 ± 6.48 nm	–	0.0003, 0.003, 0.027, 0.135, and 0.270 mg·g^−1^	Drosophila (D) melanogaster adults and larvae	SEM, TEM, DLS, laser doppler velocimetry (LDV)	Endotoxin assay, drosophila strain, exposure, and toxicity, hemocytes collection, ROS, gene expression changes, genotoxicity, antigenotoxicity, comet assay	SEM and TEM images of BNNTs revealed that the average nanotubes length was 245 ± 65.72 nm. The DLS and LDV analysis showed lower zeta potential that indicated the propensity of BNNTs to aggregates. BNNTs treated larvae increased the genotoxicity and antigenotoxicity.
Degrazia et al. [41]	PVC	–	BNNTs incorporated with bisphenol A glycerolate dimethacrylate (BisGMA) and hydroxyethyl methacrylate	0.05, 0.075, 0.1 and 0.15 wt%	Fibroblasts	FTIR, contact angle, micro tensile bond strength, failure pattern analysis	Cytotoxicity sulforhodamine B (SRB) colorimetric assay, cell viability	The successful incorporation of 0.1 wt% BNNTs into adhesive resin increased the tensile and longer stability. BNNTs treated with cells did not show any cytotoxicity.
Ferreira et al. [42]	CVD	–	BNNTs–OH– ferric oxide (Fe_3_O_4_)	0–2 µg·mL^−1^; 48 h	HeLa	XRD, TEM, XPS, vibrating sample magnetometer (VSM)	WST-8 and CCK-8 assay, internalisation tests, magneto hyperthermia assay, cell death assay (calcein-AM and PI), cell imaging	Micrograph imaging revealed a bundle of nanotubes with tube like structures. The XPS analysis showed that BNNTs consisted mostly of B and N atoms. Magnetic measurements displayed that coercivity and magnetisation were not agitated with the addition of BNNTs. The results showed excellent viability of cells treated with OH-BNNT-Fe_3_O_4_ and validated the internalisation capability of BNNTs by the cells.
Ferreira et al. [43]	CVD	–	BNNTs-OH covered with radioactive C-39 detectors	0–200 µg·mL^−1^; up to 48 h	HeLa	SEM, FTIR, XRD	WST-8, CCK-8, performance test, cells irradiation	The outcomes showed no evidence of changes in crystallinity of the material and intense solid B-N bands. No substantial differences after irradiation in the microstructures of the BNNTs compared to pure BNNTs. BNNTs had appropriate cell viability and that irradiation with a suitable flux of thermal neutron without adverse damage in the cells.
Ponraj et al. [44]	Ball milling	–	Gold nanoparticles functionalised on BNNTs and loaded with dox	30, 60, and 90 μL	DU145	TEM, XPS	Cyquant assay	Micrographs images showed long and medium BNNTs. XPS analysis displayed the BNNT surface with oxygen rate from 8 to 27.4%. The dox loaded BNNTs killed ~99% of cancer cells, which resulted in good drug carrier for cancer treatment.
Kodali et al. [45]	Commercial BNNTs	Length:0.6 to 1.6 µm	–	0–100 µg·mL^−1^	THP-1 cells, NLRP3 and c57BL/6J mice	SEM, TEM, DLS	ROS, high content epifluorescence microscopy, lysosomal membrane permeabilisation, cytokine analysis, cathepsin B and caspase 1 activity inside the cells, phagocytosis and lipopolysaccharide (LPS) functional assays	The morphology images showed BNNTs with a diameter ranging from 13–23 nm observed with a minimum agglomerate rate. BNNTs showed acute inflammation and toxicity both in vitro and in vivo condition.
Sen et al. [46]	CVD	–	Hydroxylated BNNTs modified with oligonucleotides (BNNTs- OH-oligo) and further doped with morpholino	–	MDA-MB-231-luc2	TEM, FTIR, agarose gel electrophoresis	Cell viability assay, luciferase activity	FTIR spectrum showed the B-N and –OH bands in the BNNTs and TEM images displayed some damaged nanotubes due to hydroxylation. The luciferase activity decreased when MDA-MB-231-luc2 cells were incubated with morpholino/oligo-BNNTs. The cell viability results almost similar to control.
Farshid et al. [47]	Commercial BNNTs	Length 1–2 μm and diameter ~100 nm	BNNTs reinforced propylene fumarate (PPF-BNNTs) nanocomposites	24 h	MC3T3	TEM, X-ray spectroscopy, Raman spectroscopy, sol-fraction analysis, compressive test	Presto Blue^®^ assay, LDH, Calcein-AM staining, osmolarity of degradation, cell attachment and spreading	BNNTs displayed a tubular morphology with a diameter of 100 nm and length of 1-2 µm. The spectroscopy analysis showed good bands of B-N. Furthermore, the compressive modulus increased up to 6% with the incorporation of BNNTs in PPF. BNNTs reinforced polymer nanocomposite was non-cytotoxic.
Emanet et al. [48]	CVD	Length: 5 μm; and diameter 10 nm	BNNTs-OH reinforced chitosan	Up to 7 days	HDF	SEM, TEM, fluorescent microscopy, mechanical, in vitro biodegradation	WST-1 colorimetric assay, cell proliferation and adhesion	The micrograph images showed large pores in the BNNTs-chitosan scaffolds. FTIR spectra of the BNNTs showed the -OH and B-N bands of the modified BNNTs BNNT-OH-chitosan scaffolds showed enhanced mechanical strength and reduced water absorption. The cell viability results showed that increase in viability rate over the incubation time in BNNTs-OH reinforced chitosan
Rocca et al. [49]	CVD	Length: 2 μm and diameter ~50 nm	Pectin coated BNNTs (P-BNNTs)	0 to 50 μg·mL^−1^; up to 24 h	RAW 264.7	SEM, TEM, zeta potential	WST-1 assay, quant-iT PicoGreen dsDNA assay, reactive oxygen species, annexin V-FITC apoptosis detection, cytokine detection, qRT-PCR	The results indicated that pectin coated BNNTs significantly improved the dispersibility of BNNTs. Furthermore, the micrograph analysis showed that 65% of cells positively internalised of P-BNNTs without any effects BNNTs with pectin did not show any adverse effects on cells.
Niskanen et al. [50]	Boron oxide-assisted chemical vapor deposition (BOCVD)	Length: 15 μm	BNNTs modified with isopropanol, glycine coated BNNTs loaded with curcumin	0–50 μg·mL^−1^; up to 72 h	N9 murine microglia	TEM	Confocal and non-confocal fluorescence microscopy, cellular uptake, cell viability, mitochondrial metabolic activity assay, Griess test, ELISA assay	The micrograph analysis reported that BNNTs were successfully coated with glycine and loaded with curcumin. However, the sonication resulted in shortened length and damaged some nanotubes. Non-cytotoxic.
Sen et al. [51]	CVD	–	BNNTs-OH reinforced gelatine and glucose	7 days	HDF	SEM, TEM, contact angle, tensile test, in vitro biodegradation	Cell viability, adhesion, and proliferation	The results indicated that the biodegradation amount of the scaffolds was slower with the incorporation of BNNTs. The SEM and fluorescence microscopy images showed that the BNNTs positively impacted cell adhesion and proliferation. The cells retained their own morphology and increased the proliferation rat with inclusion of BNNTs.
Li et al. [52]	CVD	Length: 1–2 μm; diameter: 80 nm	–	0–50 μg·mL^−1^; up to 14 days	MSCs	SEM, TEM, AFM, protein absorption	Cell viability	The SEM and TEM images of BNNTs showed the nanotubes of 1–2 μm length. The AFM analysis confirmed that BNNTs were uniformly distributed on the surface of piranha solution treated substrate. The protein absorption measurement indicated highest absorption ability with BNNTs on the substrate. BNNTs showed good biocompatibility with MSCs.
Diez-Pascual et al. [53]	CVD	–	Polyethylene glycol grafted BNNTs reinforced poly(propylene fumarate) (PEG-g-BNNTs-PPF)	0, 0.1, 0.5, 1.0, 2.0, 4.0 wt%; up to 24 h	HDF	FESEM, TGA, water uptake, tensile tests, antibacterial action, biodegradability, protein absorption, tribological analysis	Cell viability (alamarBlue assay)	SEM micrographs displayed a random and uniform dispersion of the PEG-g-BNNTs in the PPF. The degree of hydrophilicity, water absorption, protein absorption and biodegradability enhanced with increasing PEG-g-BNNTs content. In addition, the BNNTs nanocomposites did not show toxicity for the adhesion and growth of HDF cells.
Fernandez-Yague et al. [54]	PVC	–	Polydopamine (PD) functionalised BNNTs (PD-BNNTs)	1, 10, 30 µg·mL^−1^; up to 72 h	Osteoblasts	XPS, TEM, DLS	Live/dead assay	The TEM images indicated that the BNNTs were successfully coated with PD, and XPS analysis confirmed the presence of elemental composition of PD in BNNTs varied from BNNTs. The dispersion of PD-BNNTs in media without any precipitation was confirmed with DLS. The PD-BNNTs do not show any cytotoxic effects on cells.
Emanet et al. [55]	CVD	–	BNNTs-OH combined with glucose, lactose and starch	5 to 200 µg·mL^−1^; up to 3 days	HDF and A549	TEM, FTIR, TGA, protein interaction	Cellular uptake, ROS, cell viability, genotoxicity assay	The TEM images displayed the smooth nanotubes, and FTIR analysis confirmed the -OH and B-N bands in modified BNNTs. Furthermore, the results indicated no negative of cells treated with BNNTs.
Danti et al. [56]	–	–	BNNTs functionalised myoblast/microfibre mesh constructs	108 h	C2C12	SEM	Cellular viability, protein expression, spatial distribution, 4′-6′-diamidino-2-phenylindole staining, phalloidin-Alexa 488 stanning	Micrographs displayed the myotubes on the surface of the BNNTs. The results stated that cells were able to differentiate and to internalise upon treating with BNNTs.
Salvetti et al. [57]	CVD	Length: 10 µm; diameter: 10–80 nm	Gum Arabic coated BNNTs (GA− BNNTs)	100 or 200 µg·g^−1^; 4 and 24 h; Injected GA-BNNTs	Planarians	TEM, morphometric analysis, Inductive coupled plasma (ICP)-AES	DNA diffusion and comet assay, propidium iodide/JC1 staining, qRT-PCR, phototactic assay, analysis of mitosis	The morphological analysis demonstrated micrometres length of BNNTs, and there were no abnormalities observed after injecting GA-BNNTs into planarians. BNNTs did not induce DNA damage or apoptosis or does not show harmful effects on planarian stem cells.
Ferreira et al. [58]	CVD	Length–1 µm	BNNTs functionalised with folic acid (FA-BNNTs)	0–50 µg·mL^−1^; 1 and 3 days	HeLA	FTIR, XPS, TGA, TEM, ICP microscopy	WST-1 assay, cell uptake, lysosome staining	The FTIR analysis demonstrated bands related to B-N and C=O in Fa-BNNTs. The XPS analysis displayed strong B and N bonds in FA-BNNTs. The microscopy analysis displayed a hallow inner channel with a detailed tubular structure of nanotubes. FA-BNNTs displayed increased in cellular uptake compared to pure BNNTs.
Nakamura et al. [59]	–	–	Poly(ethyleneglycol)–1,2–distearoyl–sn–glycero–3–phosphoethanolamine (mPEG–DSPE) functionalised BNNTs (BNNTs–DSPE–PEG2000)	–	B16	–	MTT assay	BNNTs-DSPE-PEG2000 displayed antitumor effect on cells incubated over the time.
Ferreria et al. [60]	CVD	Diameter: 70 nm	Gum Arabic (GA) functionalised BNNTs (GA-BNNTs)	0–50 µg·mL^−1^; 1, 3, and 7 days	Rat MSCs	TEM, FTIR, Raman spectroscopy, DLS	Cell viability, metabolic activity, cytoskeleton conformation, differentiation of stem cells into adipocytes and osteocytes at gene and phenotype	TEM images of the BNNTs displayed hallow inner channels of nanotubes, and spectroscopy results showed the presence of B and N bands. The toxicity analysis showed that BNNTs were cyto-compatible with non-toxic effects on cells.
Li et al. [61]	CVD	–	Europium functionalised BNNTs and doped with sodium gadolinium (BNNTs@NaGdF_4_:Eu)	0–50 µg·mL^−1^; 3 and 20 h	Human LNcap prostate cancer cells	X-ray spectrometry (XRS), TEM	Cellular uptake	Micrograph images showed nanotube with inner shells coated with EU and GD. BNNTs@NaGdF_4_:Eu displayed higher cell uptake and displayed improvement of chemotherapy efficacy through magnetic fields.
Barachini et al. [62]	Ball milling and annealing	–	PLL functionalised BNNTs	0–10 µg·mL^−1^; up to 72 h	Human dental pulp stromal cells	UV–vis spectrophotometer, SEM, TEM	Cell viability, double stranded (ds-DNA) and glycosaminoglycan (GAG) contents, histological analysis	The micrographs showed that PLL-BNNTs internalised inside cytoplasm vesicles of a single DPSC. Non-cytotoxic.
Nitya et al. [63]	CVD	–	BNNTs functionalised with four surfactants: Pluronic (P123), polyethyleneimine (PEI), Pluronic (F127), and ammonium oleate (A.O.)	15.62, 31.25, 62.5, 125, 250, 500 and 1000 µg·mL^−1^; 24 h	Vero, Chang liver, MCF7 and A549	XRD, TEM, XPS	MTT assay, DNA fragmentation assay, acridine orange staining, ethidium bromide stanning	The XRD showed the hexagonal lattice of boron nitride and TEM images confirmed the presence of multiwalled BNNTs. BNNTs functionalised with four surfactants resulted in good cytocompatibility.
Ciofani et al. [64]	CVD	Length: 10 µm; diameter: 1.5 nm	GA-BNNTs	0–50 µg·mL^−1^; up to 72 h	SH-SY5Y and HUVECs	SEM	WST-1 assay, annexin V-FITC/propidium iodide (PI) apoptosis analysis, ROS, cytoskeleton analysis, immunofluorescence, qRT-PCR, detection of endothelial adhesion molecule expression	The morphology images showed that BNNTs were internalised in the cells. BNNTs with high purity about 20 µg·mL^−1^ displayed good biocompatibility.
Ferreria et al. [65]	CVD	–	BNNTs functionalised with glucosamine (GA), polyethylene glycol (PEG) 1000, and chitosan (CH)	0 to 100 µg·mL^−1^; 48 h	MRC-5	FTIR, TGA, TEM, XRD, photon correlation spectroscopy and zeta potential analysis, physical stability study, fluorescence microscope	MTT assay, ROS	The results indicated that BNNTs were successfully obtained and functionalised, achieving a standard size and dispersity considered satisfactory for in vitro studies. BNNTs functionalised with PEG and chitosan showed significant cell damage and increase cytotoxicity at higher concentration (above 50 µg·mL^−1^). However, the results stated that no considerable changes in cell morphology or increase in ROS.
Danti et al. [66]	Ball milling and annealing	–	poly-L-lysine-(PLL) coated BNNTs	0–20 µg·mL^−1^; up to 72 h	hOB	UV–vis/NIR spectrophotometer, TEM, Zeta potential distribution	MTT assay, ROS, annexin V-FITC/PI, cellular uptake, investigation of BNNTs-treated hOB cells under ultrasound irradiation, gene expression, biochemical assay, histologic analyses	The evaluation with TEM or spectroscopy confirmed that PLL-BNNTs were internalised at cytoplasm level and were noticed in membranal vesicles. The results stated PLL-BNNTs were non-cytotoxic.
Turco et al. [67]	Annealing	–	Glycol (G)-chitosan (C)-coated boron nitride nanotubes(GC-BNNTs)	0–100 µg·mL^−1^; up to 72 h	HUVECs	TEM, SEM, XRS and immunofluorescence microscopy	Cell viability, cell proliferation, surface enzyme immunoassay, cytoskeleton organisation and focal adhesions analysis, endothelial adhesion molecule expression	The SEM and TEM images displayed non-continuous nanotubes with no presence of regular stacking single units. TEM analysis indicated cellular internalisation after treating cells with GC-BNNTs. GC-BNNTs did not show adverse effects on cell biology or DNA damage, which resulted in non-cytotoxicity.
Ciofani et al. [68]	Annealing	–	Gadolinium coated BNNTs (Gd-BNNTs)	0–100 µg·mL^−1^; up to 72 h	SH-SY5Y	ICP-MS, XRS, TEM	WTS-1 assay and DNA content quantification, cell labelling using MRI experiments	The TEM images displayed defects on the nanotubes due to functionalisation. The ICP-MS and XRS confirmed the presence of B and N elements in BNNTs. Furthermore, the EDX and ICP analyses showed Gd-BNNTs as a favourable negative contrast agent. It was stated that Gd-BNNTs were biocompatible with their ability to efficiently label and distinguish in MRI images at 7 T.
Ciofani et al. [69]	Annealing	Length–500 nm	GC-BNNTs	5 and 10 mg·kg^−1^; up to 7 days; injected into the marginal ear vein of animals	New Zealand male rabbits	DLS, SEM, TEM, X-ray spectroscopy	Blood analysis, pharmacokinetic analysis, objective symptoms such as sweating, excitement, trembling, and head nodding were analysed	The morphology images displayed bamboo-like nanotubes. The DLS confirmed good dispersion in aqueous media after modification with GC.Results stated that all doses were extremely endured by the animals, with no indication of major effects.
Ciofani et al. [70]	Annealing	–	BNNTs-OH coated with 3-aminopropyl-triethoxysilane (APTES)	0–100 µg·mL^−1^; up to 24 h	NIH/3T3	Z-potential analysis, X-ray spectroscopy, SEM, TEM, XPS	WST-1 assay, ds-DNA quantification, cell internalisation analysis, actin staining	The atomic composition analysis confirmed the maximum percentage of B and N atoms present in BNNTs. The SEM/TEM images displayed nanotubes with small bundles of nanotubes. The functionalised BNNTs resulted in good cytocompatibility at higher concentration (100 µg·mL^−1^).
Soares et al. [71]	Metallic oxide-assisted chemical vapor transport	–	GC-BNNTs coated with radioelement ^99m^Tc	5 and 40 mg·kg^−1^; 10 and 30 min, 1 and 24 h; Injected intravenously into the tail of Swiss mice	Swiss mice	SEM, TGA, FTIR, photon correlation spectroscopy, zeta potential analysis	Radioactivity analysis, scintigraphy imaging biodistribution analysis	The morphology images confirmed the nanotubes coated with GC. The FTIR spectrum confirmed strong bands of B-N in BNNTs and −OH, C=H and C=H in GC-BNNTs. The TGA results displayed that BNNTs had less weight loss compared to GC-BNNTs. The in vivo distribution analysis indicated that major elimination of BNNTs by renal excretion and accumulation in the liver, spleen, and intestines.
Ciofani et al. [72]	Annealing	–	GC-BNNTs	1 mg·kg^−1^; 2, 24, and 72 h; injected into the marginal ear vein of animals	New Zealand male rabbits	FIB, TEM, AFM, Size distribution, Z-potential analysis	Blood analysis to evaluate hematic parameters and live and kidney functionality	The FIB and TEM images of BNNTs showed the presence of bamboo-like shape nanotube structures with diameter ranging between 30 and 100 nm. The AFM images revealed that nanotubes edges decorated with globular structures. Z-potential analysis demonstrated good stability of GC-BNNTs dispersion in aqueous medium. GC-BNNTs did not cause any organ failure or effects on blood parameters.
Menichetti et al. [73]	Ball milling	–	PLL-BNNTs	1–100 µg·mL^−1^; up to 72 h	SH–SY5Y	MRI experiments, UV–vis/NIR spectrophotometry	MTT assay, metabolic activity testing, cell adhesion	The PLL-BNNTsnoted at 3T showed considerable signal attenuation with increasing the concentration of BNNTs. The PLL-BNNTs compatibility in vitro at least up to 100 μg·mL^−1^.
Horvath et al. [74]	–	–	–	0.05, 1, and 2 µg·mL^−1^; up to 6 days	A549, RAW 264.7, 3T3-L1, HEK 293	SEM, TEM	Cytopathological analyses, MTT assay, FMCA assays, DNA assays	The BNNTs morphology images showed multiwalled nanotubes found in the plasmathe membrane of the cells. BNNTs induced higher toxicity in all the cell lines.
Lahiri et al. [75]	Commercial	Length–0.4–5.8 µm; diameter 10–145 nm	BNNTs reinforced hydroxyapatite (BNNTs-HA)	1, 3, and 5 days	Osteoblasts	SEM, TEM, XRD, nanoindentation, Vickers indent impression	Cell viability	The SEM/TEM images showed the nodular and cylindrical shaped BNNTs. The XRD results confirmed the hexanol lattice of B and N atoms. The composite with the highest BNNTs concentration displayed excellent mechanical properties. The BNNTs-HA did not induce any significant effects on cells.
Ciofani et al. [76]	Annealing	–	GC-BNNTs	0–100 µg·mL^−1^; up to 48 h	SH-SY5Y cells	SEM, TEM, UV–vis	MTT assay, WST-1 assay, DNA content assessment, ROS, annexin V-FITC with PI Early apoptosis detection	The SEM/TEM images showed a bamboo-shaped nanotube. Furthermore, the UV-vis spectrum confirmed strong absorption at 5.5 eV related to BNNTs. The cytotoxicity results with MTT-assay interfered the toxicity results and resulted in wrong toxicity data at low concentrationwhile WST-1 showed non-toxicity above 50 µg·mL^−1^. The results stated no significant ROS or apoptosis up to 100 µg·mL^−1^.
Lahiri et al. [77]	Commercial	–	Polylactide-polycaprolactone copolymer (PLC) reinforced with BNNTs (PLC−BNNTs)	0, 2 and 5 wt%	Osteoblasts, murine macrophages	SEM, XRD, micro-Raman spectroscopy, tensile tests	Cell viability, gene expression, nucleic acid isolation, qRT-PCR	The SEM images displayed both tubular and bamboo-shaped nanotubes. The spectroscopy strong BNNTs as well as co-polymer peaks in PLC-BNNT. The elastic modulus of PLC-BNNTs increased up to 1370% with an increase in BNNTs concentration. PLC-BNNTs incubated with cells did not increase in rate of cell death and hence resulted in non-cytotoxicity.
Ciofani et al. [78]	Ball milling and annealing	–	PLL-BNNTs	Up to 72 h	C2C12	TEM	MTT assay, live/dead assay using annexin V-FITC, metabolic activity, apoptosis detection, double stranded (ds)-DNA and protein quantification, qRT-PCR, gel electrophoresis, Western blot analysis, immunocytochemistry	The TEM images confirmed the stable dispersion with a small amount of aggregates nanotubes in dispersion agents. PLL-BNNTs did not show any difference in MyOD and Cx43 gene expression. The viability results indicated excellent cell proliferation and metabolic activity up to concentration of 10 µg·mL^−1^.
Raffa et al. [79]	Ball milling and annealing	Radius–40 nm	PLL-BNNTs	Up 24 h	SH-SY5Y	UV–vis/NIR (near-infrared) spectrophotometer, focused ion beam (FIB) microscopy, electroporation analysis	MTT assay	The UV–vis/NIR quantification reported the best and repeatability absorption of PLL-BNNTs. The microscopy images showed the bundles of nanotubes. The cells exposed to BNNTs facilitated electroporation displayed excellent cell viability, metabolism, and proliferation.
Ciofani et al. [80]	Ball milling and annealing method	–	Folic acid (FA)-PLL-BNNTs	10 µg·mL^−1^; 24 h	T98G	FIB microscopy, UV-vis spectroscopy, Z-potential	MTT assay, cellular uptake, lysosome tracking assay, Quantum dots labelling images	The FIB images showed that the FA-PLL-BNNTs could be internalised by tumour cells. The UV-vis analysis displayed firm peaks for BNNTs and PLL-BNNTs. The Z-potential evaluation showed the strong positive Z-signals for FA-PLL-BNNTs. The functionalised BNNTs indicated ability to treat malignant cerebral tumours.
Chen et al. [81]	CVD	–	–	100 mg·mL^−1^; up to 4 days	HEK 293	TEM	Cell count and cell viability using annexin V-FITC/PI assay	The microscopy images showed high purity multiwalled BNNTs. BNNTs demonstrated non-cytotoxicity.
Ciofani et al. [82,83]	Ball milling and annealing	–	Polyethyleneimine (PEI)-coated BNNTs	10 µg·mL^−1^; up to 72 h	SH-SY5Y	TEM, UV–vis/NIR spectrophotometer	Trypan blue exclusion viability assay, MTT cell proliferation assay, cell uptake, cell imaging using fluorescent microscope	The morphology images showed a bundle of nanotubes. Furthermore, the cell treated with BNNTs did not show any evidence of cell morphology changes. BNNTs treated with cells indicated no considerable effects on viability, metabolism, and cellular replication of this cell line.

**Table 3:** Summary of different synthesis methods for BNNTs.

Methods	Temperature (°C)	References
Arc-discharge	>3426.85	[3,85,87,108,109,110]
Laser ablation	1200–5000	[96,98,111,112,113]
Ball mill/annealing	1000–1300	[6,88,89,90,91,92,114,115,116,117,118,119,120,121]
Template synthesis	500–1580	[122,123,124,125,126,127,128,129]
Thermal plasma	>526.85	[84,100,101,102,130]
CVD	1100–1700	[131,132,133,134,135,136,137,138,139,140,141,142,143]
Autoclave	450–600	[107,144,145]

## Data Availability

Not applicable.
